# Motor skills and outcomes of activities and participation in children and adults born preterm without cerebral palsy: A systematic review

**DOI:** 10.1111/dmcn.70242

**Published:** 2026-03-27

**Authors:** Kari Anne I. Evensen, Silje Dahl Benum, Henriette Paulsen, Kristina Anna Djupvik Aakvik, Sindre Andre Pedersen, Tordis Ustad

**Affiliations:** ^1^ Department of Clinical and Molecular Medicine Norwegian University of Science and Technology (NTNU) Trondheim Norway; ^2^ Children's Clinic, St. Olavs Hospital, Trondheim University Hospital Trondheim Norway; ^3^ Department of Rehabilitation Science and Health Technology Oslo Metropolitan University Oslo Norway; ^4^ Trondheim University Hospital St. Olavs Hospital HF Trondheim Norway; ^5^ Department of Physiotherapy and Rehabilitation Vestfold Hospital Trust Tønsberg Norway; ^6^ Library Section for Research Support, Data and Analysis, The Medicine and Health Library, Norwegian University of Science and Technology (NTNU) Trondheim Norway; ^7^ Clinic of Rehabilitation, St. Olavs Hospital, Trondheim University Hospital Trondheim Norway

## Abstract

**Aim:**

To determine whether motor skills in children and adults born preterm (< 37 weeks’ gestation) without cerebral palsy (CP) are associated with outcomes of activities and participation within the World Health Organization's International Classification of Functioning, Disability and Health (ICF).

**Method:**

A systematic search for original articles with full text available was conducted in MEDLINE, Embase, and Web of Science. We included observational studies of children and adults born preterm without CP reporting associations between motor skills and outcomes within the Activities and Participation component of the ICF. We excluded randomized controlled trials. Screening, data extraction, and quality assessment were performed by two independent researchers.

**Results:**

Thirty‐six original articles, published from 1988 to 2025, examined 56 associations between motor skills and outcomes of activities and participation. Most associations were reported with Chapter 1 ‘Learning and applying knowledge’ and Chapter 2 ‘General tasks and demands’. Among the reported findings, 28 (50%) were positive, 10 (18%) were inconsistent, and 18 (32%) indicated no association.

**Interpretation:**

This review of the available research shows that motor skills are associated with outcomes of activities and participation in children and adults without CP born preterm. Awareness of these associations could guide assessments and interventions.

## Graphical abstract

This is the first systematic review of the associations between motor skills and outcomes of activities and participation within the World Health Organization's International Classification of Functioning, Disability and Health framework in children and adults born preterm without cerebral palsy. Motor skills were associated with attention and academic skills, behavior and general tasks, communication, daily living and physical activity, socialization, education, activity participation, and health‐related quality of life. Awareness of these associations could guide assessments and interventions in populations born preterm.

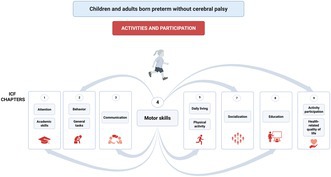

**What this paper adds**

- Motor skills were associated with several outcomes of activities and participation in children and adults born preterm without cerebral palsy.
- Most associations were reported with learning and applying knowledge, and general tasks and demands.

**Abbreviations**
BSID: Bayley Scales of Infant Development
Bayley‐III: Bayley Scales of Infant and Toddler Development, Third Edition
ELBW: extremely low birthweight
HRQoL: health‐related quality of life
LBW: low birthweight
ICF: Disability and Health
ICF‐CY: Children and Youth
Movement ABC‐2: Movement Assessment Battery for Children, Second Edition
Movement ABC: Movement Assessment Battery for Children
PDI: Psychomotor Development Index
VLBW: very low birthweight

The World Health Organization has developed the International Classification of Functioning, Disability and Health (ICF), which is based on a biopsychosocial understanding of health.^1^ Within this framework, an individual's health is the result of the interaction between several factors within body functions and structures, activities and participation, as well as personal and environmental factors. While body functions and structures relate to the body's physiology and anatomy, activities are related to the execution of tasks or actions, and participation is related to the involvement in life situations.^1^

Only a few studies have used the ICF framework in populations born preterm^2^, ^3^, ^4^, ^5^, ^6^ and most research on the consequences of preterm birth (< 37 weeks’ gestation) has traditionally focused on body functions and structures.^7^, ^8^ However, activities and participation may be more important for children's everyday life, especially as they grow older.^3^, ^4^, ^9^ Motor skills are defined as ‘tasks that require voluntary body and/or limb movement to achieve a specific goal’,^10^ and can be classified within Chapter 4 (‘Mobility’) of the ICF Activities and Participation component.^1^ Individuals born preterm are at higher risk of having motor difficulties and poorer motor skills than their peers born at term. This has been consistently reported in systematic reviews of children born very preterm (< 32 weeks’ gestation) or with very low birthweight (VLBW) (< 1500g),^11^, ^12^ even in the absence of cerebral palsy (CP)^13^, ^14^ and in children born moderate to late preterm (32 weeks to < 37 weeks’ gestation).^15^

Unlike in children with CP, where much research has focused on understanding the interplay between their activity limitations and participation,^16^, ^17^, ^18^ it is not known whether the motor consequences of preterm birth in children without CP are associated with other outcomes of activities and participation. Such outcomes may be learning and applying knowledge, general tasks and demands, communication, self‐care, domestic life, interpersonal interactions and relationships, major life areas or community, social and civic life.^1^ Knowledge of associations between motor skills and these outcomes is relevant for understanding the complex interplay of activities and participation and for potentially reducing the impact of motor difficulties on everyday life in populations born preterm. The aim of this systematic review was to determine whether motor skills are associated with other outcomes of activities and participation in children and adults without CP born preterm.

## METHOD

The study was guided by the JBI Manual for Evidence Synthesis.^19^, ^20^ We used the Preferred Reporting Items for Systematic Reviews and Meta‐Analyses (PRISMA) guidelines.^21^ The study protocol was published in the International Prospective Register of Systematic Reviews (PROSPERO) (CRD 42024584783).

### Research question

Are motor skills associated with learning and applying knowledge, general tasks and demands, communication, self‐care, domestic life, interpersonal interactions and relationships, major life areas or community, social and civic life in children and adults born preterm without CP?

### Search strategy

We performed a comprehensive literature search for observational studies investigating associations between motor skills and outcomes of activities and participation in children and adults born preterm. As we sought to examine associated outcomes as they naturally occur, we chose observational study designs because they are not limited to a single outcome of interest.^22^ The search strategy involved thesaurus and free‐text terms designed to capture records that included the three main concepts of ‘preterm birth’, ‘motor skills’, and ‘observational study design’. No restriction was put on the year of publication. The search was conducted in MEDLINE, Embase, and Web of Science, and was last updated on 2nd June 2025. The search strategies used are provided in Table S1. The reference lists of systematic reviews were also screened for potential original articles not captured in the systematic search.

### Selection process

Screening was carried out by two authors (KAIE and TU) using the following inclusion criterion: observational studies of children and adults without CP born preterm reporting an association between motor skills and outcomes within the ICF Activities and Participation component with full text available in English. As we categorized motor skills within activities and participation, we set the criterion that motor skills had to be measured with activity‐based assessment tools, such as the Movement Assessment Battery for Children (Movement ABC) or the Bayley Scales of Infant Development (BSID), and not merely neurological tests.^2^ Mapping of outcomes according to the ICF two‐level classification of activities and participation is provided in Table S2. The chapters include ‘Learning and applying knowledge’ (Chapter 1), ‘General tasks and demands’ (Chapter 2), ‘Communication’ (Chapter 3), ‘Self‐care’ (Chapter 5), ‘Domestic life’ (Chapter 6), ‘Interpersonal interactions and relationships’ (Chapter 7), ‘Major life areas’ (Chapter 8), and ‘Community, social and civic life’ (Chapter 9).^1^ The exclusion criterion was randomized controlled trials. Articles were also excluded if individuals with CP were not excluded or adjusted for in the analyses. Because CP is typically diagnosed after 1 year of age,^23^ articles that assessed motor skills or outcomes solely before the age of 1 year were excluded. Furthermore, articles that did not report associations between motor skills and outcomes within the Activities and Participation component of the ICF were excluded.

### Quality assessment

The quality of the studies included was assessed independently by two authors (KAIE and SDB) using the Newcastle–Ottawa Scale for cohort studies^24^ and adapted for cross‐sectional studies.^25^ The Newcastle–Ottawa Scale is one of the most used assessment tools to evaluate study quality worldwide.^26^ Assessment criteria were set for each of the three domains, that is, selection, comparability, and outcome (Table S3). Assessment discrepancies were resolved by discussion. The Newcastle–Ottawa Scale has a maximum score of 9 for cohort studies and 10 for cross‐sectional studies; scores less than 5 indicate a high risk of bias.^26^ We used thresholds for converting the Newcastle–Ottawa Scale scores to the standards (good, fair, and poor) of the Agency for Healthcare Research and Quality to ensure consistency across domains (Table S3).^25^

### Synthesis of the results

A qualitative synthesis of the articles included in the review was performed. The key characteristics of the articles were extracted by two authors (KAIE and TU) and are included in Table S4. Differences in outcomes of activities and participation between individuals born preterm with and without motor difficulties are presented in Tables 1 and 2. Associations between motor skills and outcomes in individuals born preterm are presented in Table 3, while associations in individuals born preterm and at term are presented in Table 4. A summary of the main findings is presented in Table 5.

**TABLE 1:** Differences in outcomes of activities and participation (ICF Chapters 1–3) between individuals born preterm with and without motor difficulties.

Citation	Preterm with MD, *n*	Preterm without MD, *n*	Cut‐off for MD	Age MD	Associated outcome	Age outcome	Mean (SD) in preterm with MD		Mean (SD) in preterm without MD		Mean difference (95% CI)		*p*	Size of association
*Chapter 1 Learning and applying knowledge*														
**EP/ELBW**														
Jeyaseelan et al.^32^	9	35	NSMDA not normal	24 months	ADHD‐RS Parent‐report	9 years	NR	NR	NR	NR	9.6	(2.0, 17.2)^a^	0.02	NA
					CRSR		NR	NR	NR	NR	9.5	(1.2, 17.8)^a^	0.03	NA
Korkman et al.^33^	32	56	Modified Touwen Motor Coordination problems	5 years	NEPSY Attention and executive functions	5 years	6.8	(3.2)	8.4	(2.7)	NR	NR	<0.05	–0.59 SD
Davis et al.^28^	20	190	Movement ABC <5 centile	8–9 years	WRAT3 Reading	8–9 years	90.6	(13.0)	100.2	(13.3)	–9.6	(–15.7, –3.5)	0.002	–0.72 SD
					WRAT Spelling		89.2	(10.3)	97.1	(10.4)	–7.8	(–12.6, –3.0)	0.002	–0.75 SD
					WRAT3 Arithmetic		81.0	(11.7)	92.9	(11.9)	–11.9	(–17.4, –6.4)	<0.001	–1.00 SD
Roberts et al.^30^	21	110	Movement ABC <5 centile	8–9 years	WRAT3 Reading	8–9 years	92.1	(20.0)	102.3	(13.2)	–10.2	(0.9, 19.7)	0.03	–0.77 SD
					WRAT Spelling		92.1	(19.5)	101.0	(12.9)	–8.9	(2.2, 15.5)	0.01	–0.69 SD
					WRAT3 Arithmetic		87.8	(18.1)	95.7	(12.6)	–7.9	(1.4, 14.5)	0.02	–0.63 SD
							***n***	**(%)**	***n***	**(%)**	**OR**	**(99% CI)**		
Bolk et al.^34^	85	144	Movement ABC‐2 <5 centile	6.5 years	Brown ADD Attention problems	6.5 years	32	(37)	20	(14)	3.38	(1.39, 8.18)^b^	<0.001	Large
Holsti et al.^31^	37	36	BOTMP <1 SD	9 years	WRAT‐R Reading	9 years	9	(25)	13	(35)	1.6	(0.6, 4.5)	NS	Small
					WRAT‐R Spelling		17	(46)	11	(31)	1.9	(0.7, 5.0)	NS	Small
					WRAT‐R Arithmetic		16	(43)	6	(16)	3.8	(1.3, 11.3)	0.02	Large
**VP/VLBW**	101	222					**Median**	**(IQR)**	**Median**	**(IQR)**				
Wocadlo and Rieger^29^			BOTMP <15 centile	8 years	NARA Reading	8 years	97	(88, 110)	101	(91, 111)	n.r.		NS	NA
					SAST Spelling		97	(88.5, 113.5)	103	(92, 119)	n.r.		<0.05	NA
					TEMA Mathematics		92	(84, 98.5)	96	(89, 106)	n.r.		<0.01	NA
*Chapter 2 General tasks and demands*														
**EP/ELBW**							**Mean**	**(SD)**	**Mean**	**(SD)**	**Mean difference (95% CI)**			
Davis et al.^28^	20	190	Movement ABC <5 centile	8–9 years	BASC Teacher adaptive functioning	8–9 years	45.8	(8.5)	48.5	(8.0)	–2.8	(–6.9, 1.4)	0.191	–0.35 SD
					BASC Teacher total behavior problems		56.6	(10.8)	49.7	(8.7)	6.9	(2.3, 11.5)	0.003	0.79 SD
					BASC Teacher externalizing problems		53.9	(10.7)	47.9	(8.7)	6.0	(1.4, 10.6)	0.010	0.69 SD
					BASC Teacher internalizing problems		53.1	(7.9)	51.4	(8.9)	1.7	(–2.8, 6.2)	0.465	0.19 SD
					BASC Parent adaptive functioning		44.8	(11.0)	50.2	(9.8)	–5.4	(–10.2, –0.6)	0.029	–0.55 SD
					BASC Parent total behavior problems		56.8	(14.0)	50.0	(12.0)	6.8	(0.9, 12.8)	0.024	0.57 SD
					BASC Parent externalizing problems		55.8	(13.3)	49.4	(11.7)	6.5	(0.7, 12.2)	0.028	0.56 SD
											**OR**	**(99% CI)**		
Bolk et al.^34^	85	144	Movement ABC‐2 <5 centile	6.5 years	SDQ Total behavioral problems	6.5 years	35	(42)	27	(19)	2.71	(1.15, 6.37)^b^	0.003	Medium
					SDQ Internalizing problems		35	(43)	37	(26)	1.85	(0.83, 4.16)^b^	0.05	Small
					SDQ Externalizing problems		29	(35)	20	(14)	2.80	(1.10, 7.12)^b^	0.005	Medium
**VP/VLBW**							**Mean**	**(SD)**	**Mean**	**(SD)**	**Mean difference (95% CI)**			
Spittle et al.^42^	28	79	Movement ABC‐2 ≤16 centile	4–5 years	SDQ Total difficulties	4–5 years	11.4	(7.5)	9.30	(5.1)	–0.1	(–3.3, 2.8)^c^	0.92	–0.02 SD
							***n***	**(%)**	***n***	**(%)**	**OR**	**(95% CI)**		
Moreira et al.^43^	39	61	Movement ABC‐2 <15 centile	8–10 years	SDQ Deficient behavior score	8–10 years	13	(33.3)	18	(29.5)	NR	NR	0.45	NA
*Chapter 3 Communication*														
**EP/ELBW**							**Mean**	**(SD)**	**Mean**	**(SD)**				
Korkman et al.^33^	32	56	Modified Touwen Motor Coordination problems	5 years	NEPSY Language	5 years	7.4	(1.9)	8.4	(1.7)			<0.05	0.59 SD
							***n***	**(%)**						
Charkaluk et al.^49^	230	2194	ASQ‐2 Gross Motor below threshold	2 years	Lexicon size <10p EP	2 years	38	(28.1)					0.06^d^	NA
					Lexicon size <10p VP		76	(15.7)					<0.001^d^	NA
					Lexicon size <10p MP		16	(9.7)					0.03^d^	NA
	260	2164	ASQ‐2 Fine Motor below threshold		Lexicon size <10p EP		27	(20)					0.04^d^	NA
					Lexicon size <10p VP		90	(18.6)					<0.001^d^	NA
					Lexicon size <10p MP		31	(18.8)					0.005^d^	NA

*Note*: *n*, number; *p*, *p*‐value.

Abbreviations: ADD, attention deficit disorder; ADHD‐RS, Attention Deficit Hyperactivity Disorder Rating Scale; ASQ‐2, Ages & Stages Questionnaire, Second Edition; BASC, Behavior Assessment System for Children; BOTMP, Bruininks‐Oseretsky Test of Motor Proficiency; CI, confidence interval; CRSR, Conners’ Rating Scale Revised‐Long Form; ELBW, extremely low birthweight; EP, extremely preterm; ICF, International Classification of Functioning, Disability and Health; IQR, interquartile range; MD, motor difficulties; Movement ABC, Movement Assessment Battery for Children; Movement ABC‐2, Movement Assessment Battery for Children, Second Edition; MP, moderately preterm; NA, not applicable; NARA, Neale Analysis of Reading Ability; NEPSY, Developmental Neuropsychological Assessment; NR, not reported; NS, not significant; NSMDA, Neurological, Sensory, Motor, Developmental Assessment; OR, odds ratio; SAST, South Australian Spelling Test; SD, standard deviation; SDQ, Strengths and Difficulties Questionnaire; TEMA, Test of Early Mathematics Ability; VLBW, very low birthweight; VP, very preterm; WRAT3, Wide Range Achievement Test, Third Edition; WRAT‐R, Wide Range Achievement Test, Revised.

^a^ Adjusted for sex, gestational age, birthweight, public versus private admission, maternal age, and education.

^b^ Adjusted for sex, gestational age, age at assessment, mother's country of birth (Nordic/non‐Nordic), and clustering effects of twins.

^c^ Adjusted for sex, gestational age, birthweight z‐score, oxygen at 36 weeks, necrotizing enterocolitis, intraventricular hemorrhage grade III/IV, and social risk.

^d^ Adjusted for sex, gestational age, small for gestational age, language spoken at home, maternal parity, maternal educational level, and multiple births.

**TABLE 2:** Differences in outcomes of activities and participation (ICF Chapters 5–9) between individuals born preterm with and without motor difficulties.

Citation	Preterm with MD, *n*	Preterm without MD, *n*	Cut‐off for MD	Age MD	Associated outcome	Age outcome	Mean (SD) in preterm with MD		Mean (SD) in preterm without MD		Mean difference (95% CI)		*p*	Size of association
*Chapter 5 Self‐care*														
**EP/ELBW**							**OR**	**(95% CI)**						
Engan et al.^53^	21	170	Movement ABC <5 centile	5 years	Leisure time PA ≤1 day/week	11 years	1.33	(0.45, 3.90)^a^	Ref				0.601	Small
					Less VPA		5.27	(2.00, 13.84)^b^	Ref				0.001	Large
**VP/VLBW**							**Mean**	**(SD)**	**Mean**	**(SD)**	**Mean difference** **(95% CI)**			
Spittle et al.^42^	28	79	Movement ABC‐2 ≤16 centile	4–5 years	PEDI‐CAT Activities	4–5 years	48.60	(8.1)	52.40	(5.8)	–3.2	(–6.0, –0.50)^c^	0.02	–0.55 SD
							***n***	**(%)**	***n***	**(%)**	**OR**	**(95% CI)**		
Maggi et al.^54^	62	62	Movement ABC‐2 ≤5 centile	4 years	PEDI‐FS	4 years	NR	NR	NR	NR	NR	NR	0.897	NA
					PEDI‐CA		NR	NR	n.r.	n.r.	NR	NR	0.697	NA
							***B***	**(95% CI)**				β		
Proulx et al.^56^	62 in total sample		Movement ABC‐2 ≤16 centile	12–20 years	MVPA	12–20 years	18.64	(3.93, 33.36)^d^	Ref			0.26^d^	0.014	Small
*Chapter 7 Interpersonal interaction and relationships*														
**EP/ELBW**											**OR**	**(99% CI)**		
Bolk et al.^34^	85	144	Movement ABC‐2 <5 centile	6.5 years	SDQ Peer relations	6.5 years	15	(18)	20	(14)	1.10	(0.37, 3.30)^e^	0.81	Small
					FTF Social skills		22	(28)	24	(18)	1.46	(0.59, 3.63)^e^	0.28	Small
**VP/VLBW**							**Mean**	**(SD)**	**Mean**	**(SD)**	**Mean difference** **(95% CI)**			
Spittle et al.^42^	28	79	Movement ABC‐2 ≤16 centile	4–5 years	PedsQL Social functioning	4–5 years	70.50	(17.9)	79.90	(19.1)	–9.3	(–18.8, 0.2)^c^	0.05	–0.49 SD
					SDQ Peer problems		2.0	(2.29)	1.20	(1.4)	0.60	(–0.3, 1.4)^c^	0.17	0.43 SD
							***r***	***p***	***r***	***p***				
Helin et al.^57^	18	141	Movement ABC‐2 ≤5 centile	11 years	PNDLS Social loneliness	11 years	0.3	0.3	–0.1	0.4				Small
					PNDLS Emotional loneliness		–0.3	0.2	0.0	0.6				Small
					MASCS Cooperation skills		–0.2	0.4	0.0	0.7				Small
					MASCS Empathy		–0.3	0.3	0.1	0.5				Small
*Chapter 8 Major life areas*														
**EP/ELBW**							**OR**	**(95% CI)**					***p***	
Wiingreen et al.^59^	49	122	Movement ABC‐2 ≤5 centile	5 years	School difficulties	18 years	2.0	(0.7, 3.2)^f^	Ref				NS	Medium
	45	122	Movement ABC‐2 6–15 centile				1.7	(0.7, 4.0)^f^	Ref				NS	Small
**VP/VLBW**														
Verkerk et al.^60^	117 in total sample		VMI‐6 Motor Coordination <1SD	3.5 years	Educational provision	5.5 years	4.6	(1.9, 11.0)^g^					<0.001	Large
							***n***	**(%)**	***n***	**(%)**				
Marlow et al.^61^	35	16	TOMI >5 points	6 years	School difficulties (in 2–3 subjects)	8 years	12	(92)	1	(8)			NR	NA
Wocadlo and Rieger^29^	101	222	BOTMP <15 centile	8 years	Literacy under‐achievement	8 years	40	(39.6)	54	(24.3)			<0.01	NA
					Numeracy under‐achievement		41	(40.6)	58	(26.1)			<0.05	NA
					Literacy and numeracy underachievement		26	(25.7)	34	(15.4)			<0.05	NA
					Remedial education		63	(62.4)	98	(44.1)			<0.01	NA
Moreira et al.^43^	39	61	Movement ABC‐2 <15 centile	8–10 years	TDE Inferior academic performance	8–10 years	17	(43.6)	14	(23.0)			0.09	NA
					Not able to follow school activities at same level as others		14	(35.9)	11	(18.3)			0.08	NA
*Chapter 9 Community, social, and civic life*														
**EP/ELBW**							**OR**	**(95% CI)**						
Engan et al.^53^	21	170	Movement ABC	5 years	No organized sports activities	11 years	1.66	(0.61, 4.50)^a^	Ref				0.320	Small
**VP/VLBW**							***n***	**(%)**	***n***	**(%)**				
Wocadlo and Rieger^29^	101	222	BOTMP <15 centile	8 years	No sporting activity after school hours	8 years	54	(53.5)	87	(39.2)			<0.05	NA
							**Mean**	**(SD)**	**Mean**	**(SD)**	**Mean difference (95% CI)**			
Spittle et al.^42^	28	79	Movement ABC‐2 ≤16 centile	4–5 years	PedsQL Physical	4–5 years	79.30	(16.8)	82.40	(19.3)	–4.7	(–14.4, 4.9)^c^	NS	0.34 SD
					PedsQL Psychosocial		70.30	(14.0)	77.10	(14.7)	–6.3	(–14.8, 2.2)^c^	NS	0.14 SD
Uusitalo et al.^63^	18	142	Movement ABC‐2 ≤5 centile	11 years	17D	11 years	0.93	NR	0.96	NR	–0.03	NR	0.03	NA
							***β***	**(95% CI)**						
Aubert et al.^62^	216	330	Movement ABC‐2 ≤5 centile	5 years	PedsQL Physical	5 years	–11.9	(–16.1, –7.8)^h^	Ref				NR	NA
					PedsQL Psychosocial		–7.4	(–10.3, –4.5)^h^	Ref				NR	NA
					PedsQL Total		–9.1	(–12.0, –6.1)^h^	Ref				NR	NA
	165	330	Movement ABC‐2 ≤15 centile		PedsQL Physical		–5.4	(–9.1, –1.6)^h^	Ref				NR	NA
					PedsQL Psychosocial		–4.9	(–7.6, –2.1)^h^	Ref				NR	NA
					PedsQL Total		–5.0	(–7.7, –2.3)^h^	Ref				NR	NA

*Note*: *n*, number; *p*, *p*‐value.

Abbreviations: BOTMP, Bruininks‐Oseretsky Test of Motor Proficiency; CI, confidence interval; ELBW, extremely low birthweight; EP, extremely preterm; FTF, Five to Fifteen Questionnaire; ICF, International Classification of Functioning, Disability and Health; MASCS, Multisource Assessment of Children's Social Competence Scale; MD, motor difficulties; Movement ABC, Movement Assessment Battery for Children; Movement ABC‐2, Movement Assessment Battery for Children, Second Edition; MVPA, moderate to vigorous physical activity; NA, not applicable; NR, not reported; NS, not significant; OR, odds ratio; PA, physical activity; PEDI‐CA, Pediatric Evaluation of Disability Inventory, Caregiver Assistance; PEDI‐CAT, Pediatric Evaluation of Disability Inventory, Computer Adaptive Test; PEDI‐FS, Pediatric Evaluation of Disability Inventory, Functional Skills; PedsQL, Pediatric Quality of Life Inventory; PNDLS, Peer Network and Dyadic Loneliness Scale; SD, standard deviation; SDQ, Strengths and Difficulties Questionnaire; TDE, Teste de Desempenho Escolar; TOMI, Test of Motor Impairment; VLBW, very low birthweight; VMI‐6, Beery‐Buktenica Developmental Test of Visual‐Motor Integration, Sixth Edition; VP, very preterm; VPA, vigorous physical activity; 17D, 17‐Dimensional Illustrated Questionnaire.

^a^ Adjusted for maternal education.

^b^ Adjusted for use of asthma medication at 5 years of age.

^c^ Adjusted for sex, gestational age, birth weight z‐score, oxygen at 36 weeks, necrotizing enterocolitis, intraventricular hemorrhage grade III/IV, and social risk.

^d^ Adjusted for sex, age, health problems (including cerebral palsy), hyperactivity behavior.

^e^ Adjusted for sex, gestational age, age at assessment, mother's country of birth (Nordic/non‐Nordic), and clustering effects of twins.

^f^ Adjusted for intelligence quotient, motor performance, sex, multiple births, and maternal educational level.

^g^ Adjusted for cognitive measures, executive function, and behavior.

^h^ Adjusted for sex, child age, maternal age and parity at child's birth, maternal country of birth, parental cohabiting status, maternal educational level, and household unemployment status.

**TABLE 3:** Associations between motor skills and outcomes of activities and participation in individuals born preterm.

Citation	Born preterm, *n*	Measure assessing motor skills	Age motor skills	Associated outcome	Age outcome	Measure of association in the group born preterm		*p*	Size of association
*Chapter 1 Learning and applying knowledge*									
**EP/ELBW**						***B***	**SE**		
Lowe et al.^35^	1074	Bayley‐III Motor	22–26 months	CBCL Attention‐deficit/ hyperactivity problems	22–26 months	0.59	0.69^a^	0.39	NA
*Chapter 2 General tasks and demands*									
**EP/ELBW**									
Lowe et al.^35^	1074	Bayley‐III Motor	22–26 months	CBCL Internalizing	22–26 months	*−*2.54	0.48^a^	< 0.001	NA
				CBCL Externalizing		0.25	0.45^a^	0.58	NA
**VP/VLBW**						***β/B***	**(95% CI)**		
Wollum et al.^44^	24	BSID PDI	1 year	ASR Mean adaptive	26 years	0.09/0.03	*(−*0.1 to 0.2)	0.787	Small
				GAF Function		*−*0.05/0.02	*(−*0.3 to 0.5)	0.848	Small
	25	PDMS Eye–Hand	5 years	ASR Mean adaptive		0.09/0.1	*(−*0.3 to 0.6)	0.635	Small
				GAF Function		0.45/1.1	*(−*0.2 to 2.5)	0.305	Medium
	24	PDMS Balance	5 years	ASR Mean adaptive		0.27/0.4	*(−*0.6 to 0.9)	0.450	Small
				GAF Function		0.22/0.8	*(−*2.0 to 3.4)	0.659	Small
	24	PDMS Locomotor	5 years	ASR Mean adaptive		0.20/0.1	*(−*0.2 to 0.4)	0.558	Small
				GAF Function		0.37/0.5	*(−*0.4 to 1.3)	0.390	Small
	45	Movement ABC	14 years	ASR Mean adaptive		*−*0.08/*−*0.1	*(−*0.3 to 0.3)	0.650	Small
				GAF Function		*−*0.40/*−*0.7	*(−*1.5 to 0.4)	0.080	Small
**MLP/LBW**						***r***			
Blasco et al.^46^	178	Bayley‐III Fine Motor	10 months	DMQ18 General competence	10 months	0.232		<0.01	Small
		Bayley‐III Gross Motor		DMQ18 General competence		0.201		<0.01	Small
	55	Bayley‐III Fine Motor	26.7 months	DMQ18 General competence	26.7 months	0.241		NS	Small
		Bayley‐III Gross Motor		DMQ18 General competence		0.277		NS	Small
						***r***_***s***_			
Jongmans et al.^47^	156	Movement ABC	76 months	Rutter Teacher's Scale	76 months	0.27		0.004	Small
				Rutter Parents’ Scale		0.08		0.321	Small
*Chapter 3 Communication*									
**VP/VLBW**						***r***			
Panceri et al.^51^	95	Bayley‐III Motor	4 months	Bayley‐III Language	36 months	0.006		NS	Small
		Bayley‐III Motor	8 months	Bayley‐III Language	36 months	0.471		≤ 0.001	Medium
		Bayley‐III Motor	12 months	Bayley‐III Language	36 months	0.520		≤ 0.001	Medium
Howe et al.^52^	126	AIMS	12 months	VABS‐C Communication	5 years	NR		NS	NA
		BSID PDI	12 months	VABS‐C Communication		NR		NS	NA
		BSID PDI	18 months	VABS‐C Communication		NR		NS	NA
		BSID PDI	24 months	VABS‐C Communication		NR		NS	NA
*Chapter 5 Self‐care*									
**VP/VLBW**						***B***	**(95% CI)**		
FitzGerald et al.^55^	123	Movement ABC‐2 Total	4–5 years	Total physical activity (hours)	4–5 years	0.13	*(−*0.26 to 0.52)^b^	0.52	NA
				Stepping physical activity (hours)		*−*0.18	*(−*1.11 to 0.75)^b^	0.70	NA
				Fast steps (*n*)		*−*0.07	*(−*0.24 to 0.10)^b^	0.42	NA
				Stationary time (hours)		0.19	*(−*0.33 to 0.71)^b^	0.48	NA
				Unstructured physical activity (hours)		*−*0.11	(*−*0.72 to 0.50)^b^	0.72	NA
				Structured physical activity (hours)		0.37	*(−*0.40 to 1.13)^b^	0.35	NA
		Movement ABC‐2 Manual Dexterity		Total physical activity (hours)		*−*0.04	*(−*0.54 to 0.47)^b^	0.88	NA
				Stepping physical activity (hours)		*−*0.46	(*−*1.38 to 0.45)^b^	0.32	NA
				Fast steps (*n*)		*−*0.11	*(−*0.27 to 0.05)^b^	0.18	NA
				Stationary time (hours)		0.32	*(−*0.18 to 0.81)^b^	0.21	NA
				Unstructured physical activity (hours)		*−*0.50	*(−*1.19 to 0.20)^b^	0.16	NA
				Structured physical activity (hours)		*−*0.06	*(−*0.90 to 0.79)^b^	0.89	NA
		Movement ABC‐2 Aiming & Catching		Total physical activity (hours)		0.30	*(−*0.13 to 0.72)^b^	0.17	NA
				Stepping physical activity (hours)		0.29	(*−*0.65 to 1.23)^b^	0.48	NA
				Fast steps (*n*)		*−*0.03	(*−*0.19 to 0.12)^b^	0.66	NA
				Stationary time (hours)		0.01	*(−*0.52 to 0.55)^b^	0.96	NA
				Unstructured physical activity (hours)		0.19	*(−*0.37 to 0.75)^b^	0.50	NA
				Structured physical activity (hours)		0.72	0.09 to 1.36^b^	0.03	NA
		Movement ABC‐2 Balance		Total physical activity (hours)		0.10	*−*0.22 to 0.43^b^	0.53	NA
				Stepping physical activity (hours)		0.03	(*−*0.76 to 0.82)^b^	0.95	NA
				Fast steps (*n*)		*−*0.003	*(−*0.16 to 0.15)^b^	0.97	NA
				Stationary time (hours)		0.05	(*−*0.41 to 0.52)^b^	0.82	NA
				Unstructured physical activity (hours)		0.11	*(−*0.38 to 0.59)^b^	0.67	NA
				Structured physical activity (hours)		*−*0.09	*−*0.75 to 0.58^b^	0.80	NA
Howe et al.^52^	126	AIMS	12 months	VABS‐C Daily Living Skills	5 years	0.327^c^		< 0.01	Small
		BSID PDI	12 months	VABS‐C Daily Living Skills		NR		NS	NA
		BSID PDI	18 months	VABS‐C Daily Living Skills		NR		NS	NA
		BSID PDI	24 months	VABS‐C Daily Living Skills		NR		NS	NA
*Chapter 7 Interpersonal interaction and relationships*									
**VP/VLBW**						***B***			
Howe et al.^52^	126	AIMS	12 months	VABS‐C Socialization	5 years	NR		NS	NA
		BSID PDI	12 months	VABS‐C Socialization		NR		NS	NA
		BSID PDI	18 months	VABS‐C Socialization		NR		NS	NA
		BSID PDI	24 months	VABS‐C Socialization		0.245^c^		<0.05	NA
**MLP/LBW**						***r***			
Blasco et al.^46^	178	Bayley‐III Fine Motor	10 months	DMQ18 Social persistence adults	10 months	0.005		NS	Small
				DMQ18 Social persistence children		0.017		NS	Small
			26.7 months	DMQ18 Social persistence adults	26.7 months	0.074		NS	Small
				DMQ18 Social persistence children		0.253		NS	Small
		Bayley‐III Gross Motor	10 months	DMQ18 Social persistence adults	10 months	*−*0.007		NS	Small
				DMQ18 Social persistence children		0.003		NS	Small
			26.7 months	DMQ18 Social persistence adults	26.7 months	0.177		NS	Small
				DMQ18 Social persistence children		0.121		NS	Small
*Chapter 9 Community, social and civic life*									
**VP/VLBW**						***β/B***	**(95% CI)**		
Wollum et al.^44^	24	BSID PDI	1 year	SF‐36 Physical	28 years	0.33/0.1	*(−*0.2 to 0.3)	0.196	Small
				SF‐36 Mental		0.12/0.1	(*−*0.1 to 0.5)	0.653	Small
	25	PDMS Eye–Hand	5 years	SF‐36 Physical		0.52/0.6	(0.2 to 1.1)	0.008	Medium
				SF‐36 Mental		0.17/0.3	*(−*0.6 to 1.0)	0.608	Small
	24	PDMS Balance		SF‐36 Physical		0.51/0.7	*(−*0.1 to 1.2)	0.026	Medium
				SF‐36 Mental		0.08/0.2	(*−*1.2 to 1.1)	0.735	Small
	24	PDMS Locomotor		SF‐36 Physical		0.48/0.3	*−*0.1 to 0.5	0.045	Medium
				SF‐36 Mental		0.14/0.1	*(−*0.3 to 0.5)	0.592	Small
	45	Movement ABC	14 years	SF‐36 Physical		*−*0.21/0.2	(*−*0.5 to 0.1)	0.212	Medium
				SF‐36 Mental		*−*0.55/*−*0.7	*(−*1.0 to *−*0.1)	0.002	Medium

*Note*: *β*, standardized beta; *B*, unstandardized beta; *p*, *p*‐value; *n*, number; *r*, Pearson's correlation coefficient; *r*_*s*_, Spearman's rank correlation coefficient.

Abbreviations: AIMS, Alberta Infant Motor Scale; ASR, adult self‐report; Bayley‐III, Bayley Scales of Infant and Toddler Development, Third Edition; BSID, Bayley Scales of Infant Development; CBCL, Child Behavior Checklist; CI, confidence interval; DMQ18, Revised Dimensions of Mastery Questionnaire; ELBW, extremely low birthweight; EP, extremely preterm; GAF, Global Assessment of Functioning; LBW, low birthweight; MLP, moderate to late preterm; Movement ABC, Movement Assessment Battery for Children; Movement ABC‐2, Movement Assessment Battery for Children, Second Edition; NA, not applicable; NR, not reported; NS, not significant; PDI, Psychomotor Development Index; PDMS, Peabody Developmental Motor Scales; SF‐36, 36‐Item Short Form Health Survey; VABS‐C, Vineland Adaptive Behavior Scales, Chinese version; VLBW, very low birthweight; VP, very preterm.

^a^ Adjusted for center, sex, gestational age, small forgestational age, age at testing, maternal medical insurance, ethnicity, bronchopulmonary dysplasia, intraventricular haemorrhage/periventricular leukomalacia, late‐onset sepsis, retinopathy of prematurity, necrotizing enterocolitis, and patent ductus arteriosus.

^b^ Adjusted for sex, social risk and the clustering effect of multiple births.

^c^ Adjusted for sex, small for gestational age, medical complications, and maternal education.

**TABLE 4:** Associations between motor skills and outcomes of activities and participation in individuals born preterm and at term.

Study	Born preterm, *n*	Control, *n*	Measure assessing motor skills	Age motor skills	Associated outcome	Age outcome	Measure of association in the group born preterm		Measure of association in the control group		Size of association
*Chapter 1 Learning and applying knowledge*											
							**Interaction term of motor skills by group**				
**EP/ELBW**							***B/β***	**SE**		***p***	
Poole et al.^36^	151	145	ADC	Childhood (reported 29–36 years)	CBCL Parent‐report attention problems	8 years	*−*0.11	0.14^a^		NS	NA
	151	145			CBCL Teacher‐report attention problems	8 years	*−*0.04	0.18^a^		NS	NA
	142	133			YASR Attention problems	22–26 years	*−*0.29	0.11^a^		< 0.01	NA
	100	89			YASR Attention problems	29–36 years	*−*0.21	0.11^a^		< 0.05	NA
	142	133			Barkley ADHD total score	22–26 years	*−*0.57	0.26^a^		< 0.05	NA
**VP/VLBW**							***β***	**(95% CI)**	***β***	**(95% CI)**	
Brown et al.^37^	197	69	Movement ABC‐2 Aiming & Catching	7 years	TEA Selective attention	7 years, 13 years	0.26	(0.12 to 0.41)^b^	*−*0.85	(*−*0.31 to 0.14)^b^	Small
			Movement ABC‐2 Manual Dexterity		TEA Sustained attention		0.53	(0.38 to 0.68)^b^	0.05	*(−*0.31 to 0.42)^b^	Medium
			Movement ABC‐2 Aiming & Catching		TEA Divided attention		No group‐motor interaction				NA
			Movement ABC‐2 Manual Dexterity		SDQ Behavioural attention		No group‐motor interaction				NA
**MLP/LBW**							***β***	***B***	**(95% CI)**		
Bogičević et al.^39^	88	83	Bayley‐III Motor	24 months	CBCL Attention problems	6 years	0.14	0.07	(*−*0.02 to 0.15)^c^	Ref.	Small
							***z***	***p***			
Martel et al.^38^	473	350	GP Motor speed	6 years	CBCL Inattention/hyperactivity	6 years	*−*4.50	*p* < 0.05	Partial mediation of birthweight effect		NA
			GP Motor coordination				6.16	*p* < 0.05	Partial mediation of birthweight effect		NA
							*B*	*p*			
Hasler and Akshoomoff^40^	58	29	Movement ABC‐2	5 years	TEMA‐3	5 years	0.326^d^	0.516	No mediation of birthweight effect		NA
*Chapter 2 General tasks and demands*											
**EP/ELBW**							***β/B***	**95% CI**	***B/β***	***p***	
Danks et al.^41^	48	44	Movement ABC	11–13 years	CBCL Total problems	11–13 years	0.28/2.17	0.36 to 3.98^e^	No group‐motor interaction		Small
**MLP/LBW**							**OR**	**(95% CI)**	**OR**	**(95% CI)**	
van Dokkum et al.^48^	769	472	Age at walking	NA	ASQ Problem‐solving less than *−*2SD	4 years	1.06	(0.97 to 1.15)^f^	0.93	(0.71 to 1.23)^f^	Small
							***r***	***p***	***r***	***p***	
Greenberg and Crnic^45^	30	40	BSID PDI	24 months	Composite of infant temperament and behaviour during free play and structured interaction	8 months	0.47	*p* < 0.05	NR	NS	Medium
							***β***	***B***	**(95% CI)**		
Bogičević et al.^39^	88	83	Bayley‐III Motor	24 months	CBCL Internalizing	6 years	0.03	NR	NS	Ref.	Small
					CBCL Externalizing			0.03	*(−*0.12 to 0.18)^c^	Ref.	Small
*Chapter 3 Communication*											
**EP/ELBW**							***r***_***s***_	***p***	***r***_***s***_	***p***	
Benassi et al.^50^	20	20	Bayley‐III Fine motor	12 months	Requesting	12 months	*−*0.039	0.871	*−*0.045	0.851	Small
					Pointing		0.631	0.003	0.064	0.787	Small
					Showing		*−*0.034	0.886	0.089	0.710	Small
					Giving		0.362	0.117	0.446	0.049	Medium
					Conventional		*−*0.357	0.112	0.290	0.215	Small
					Representational		0.623	0.003	0.090	0.705	Small
			Bayley‐III Gross motor		Requesting		0.093	0.697	*−*0.114	0.631	Small
					Pointing		0.398	0.082	*−*0.200	0.397	Small
					Showing		0.001	0.356	*−*0.089	0.709	Small
					Giving		0.131	0.758	0.359	0.120	Small
					Conventional		0.127	0.446	*−*0.473	0.035	Medium
					Representational		0.388	0.320	*−*0.149	0.531	Small
**MLP/LBW**							**OR**	**95% CI**	**OR**	**95% CI**	
van Dokkum et al.^48^	769	472	Age of walking	NA	ASQ Communication less than *−*2SD	4 years	1.06	0.99 to 1.13^f^	1.14	0.97 to 1.34^f^	Small
*Chapter 5 Self‐care*											
**MLP/LBW**											
van Dokkum et al.^48^	769	472	Age at walking	NA	ASQ Personal‐Social less than *−*2SD	4 years	1.10	1.01 to 1.18^f^	1.33	1.07 to 1.65^f^	Small
*Chapter 7 Interpersonal interaction and relationships*											
**VP/VLBW**							***β***	**95% CI**	***β***	**95% CI**	
Liu et al.^58^	234	226	TOMI	6 years	PSPCSA, SCA	6–26 years	0.03	0.01 to 0.07^g^	Partial mediation (19%) of birthweight effect on decreasing social self‐concept over time		Small

*Note*: *β*, standardized beta; *B*, unstandardized beta; *p*, *p*‐value; *n*, number; *r*, Pearson's correlation coefficient; *r*_*s*_, Spearman's rank correlation coefficient; *z*, Sobel test.

Abbreviations: ADC, Adult Developmental Coordination Disorders/Dyspraxia Checklist; ADHD, attention‐deficit/hyperactivity disorder; ASQ, Ages & Stages Questionnaire; Bayley‐III, Bayley Scales of Infant and Toddler Development, Third Edition; BSID, Bayley Scales of Infant Development; CBCL, Child Behavior Checklist; CI, confidence interval; ELBW, extremely low birthweight; EP, extremely preterm; GP, Grooved Pegboard test; LBW, low birthweight; MLP, moderate to late preterm; Movement ABC, Movement Assessment Battery for Children; Movement ABC‐2, Movement Assessment Battery for Children, Second Edition; NA, not applicable; NR, not reported; NS, not significant; OR, odds ratio; PDI, Psychomotor Development Index; PSPCSA, Pictorial Scale of Perceived Competence and Social Acceptance for Young Children; SCA, Nicholls’ Self‐Concept of Attainment; SDQ, Strengths and Difficulties Questionnaire; SE, standard error; TEA, Test of Everyday Attention for Children; TEMA‐3, Test of Early Mathematics Ability, Third Edition; TOMI, Test of Motor Impairment; VLBW, very low birthweight; VP, very preterm; YASR, Young Adult Self Report.

^a^ Adjusted for neurosensory impairments (including cerebral palsy).

^b^ Adjusted for sex and social risk.

^c^ Adjusted for group, maternal education, and cognitive and language skills.

^d^ Adjusted for cognitive measures.

^e^ Adjusted for sex, small for gestational age, group, and socioeconomic status.

^f^ Adjusted for sex, small for gestational age, maternal educational level, and ethnicity.

^g^ Adjusted for sex and socioeconomic status.

**TABLE 5:** Summary of associations between motor skills and outcomes of activities and participation by degree of preterm birth (*n* = 56).

	Chapter 1 Learning and applying knowledge (*n* = 13)		Chapter 2 General tasks and demands (*n* = 12)		Chapter 3 Communication (*n* = 8)	Chapter 5 Self‐care (*n* = 7)		Chapter 7 Interpersonal interaction and relationships (*n* = 5)	Chapter 8 Major life areas (*n* = 5)	Chapter 9 Community, social and civic life (*n* = 6)	
	Attention	Academic skills	Behaviour	General tasks	Communication	Daily living	Physical activity	Socialization	Education	Activity participation	HRQoL
EP/ELBW	Jeyaseelan et al.^32^ ^,^ ^a^	Holsti et al.^31^ ^,^ ^c^	Lowe et al.^36^ ^,^ ^a^		Korkman et al.^33^ ^,^ ^a^		Proulx et al.^56^ ^,^ ^a^	Bolk et al.^34^ ^,^ ^a^	Wiingreen et al.^59^ ^,^ ^a^	Engan et al.^53^ ^,^ ^a^	
	Korkman et al.^33^ ^,^ ^c^	Davis et al.^28^ ^,^ ^a^	Bolk et al.^34^ ^,^ ^a^		Charkaluk et al.^49^ ^,^ ^b^		Engan et al.^53^ ^,^ ^a^	Helin et al.^57^ ^,^ ^a^			
	Bolk et al.^34^ ^,^ ^a^	Roberts et al.^30^ ^,^ ^c^	Davis et al.^28^ ^,^ ^a^		Benassi et al.^50^ ^,^ ^c^						
	Poole et al.^36^ ^,^ ^b^		Danks et al.^41^ ^,^ ^a^								
	Lowe et al.^35^ ^,^ ^a^										
VP/VLBW	Brown et al.^37^ ^,^ ^a^	Wocadlo and Rieger^29^ ^,^ ^c^	Spittle et al.^42^ ^,^ ^a^	Wollum et al.^44^ ^,^ ^a^	Charkaluk et al.^49^ ^,^ ^b^	Spittle et al.^42^ ^,^ ^a^	FitzGerald et al.^55^ ^,^ ^a^	Howe et al.^52^ ^,^ ^a^	Verkerk et al.^60^ ^,^ ^b^	Wocadlo and Rieger^29^ ^,^ ^c^	Aubert et al.^62^ ^,^ ^a^
			Moreira et al.^43^ ^,^ ^b^		Panceri et al.^51^ ^,^ ^b^	Howe et al.^52^ ^,^ ^a^		Spittle et al.^42^ ^,^ ^a^	Marlow et al.^61^ ^,^ ^a^		Uusitalo et al.^63^ ^,^ ^a^
					Howe et al.^52^ ^,^ ^a^	Maggi et al.^54^ ^,^ ^b^			Wocadlo and Rieger^29^ ^,^ ^c^		Wollum et al.^44^ ^,^ ^a^
									Moreira et al.^43^ ^,^ ^b^		Spittle et al.^42^ ^,^ ^a^
MLP/LBW	Martel et al.^38^ ^,^ ^a^	Hasler and Akshoomoff^40^ ^,^ ^a^	Greenberg and Crnic^45^ ^,^ ^c^	Blasco et al.^46^ ^,^ ^c^	Charkaluk et al.^49^ ^,^ ^b^	Van Dokkum et al.^48^ ^,^ ^a^		Blasco et al.^46^ ^,^ ^c^			
	Bogičević et al.^39^ ^,^ ^a^		Jongmans et al.^47^ ^,^ ^c^	Van Dokkum et al.^48^ ^,^ ^a^	Van Dokkum et al.^48^ ^,^ ^a^						
			Bogičević et al^39^ ^,^ ^a^								

*Note*: Dark grey indicates positive associations; light grey indicates inconsistent findings; white indicates no association.

Abbreviations: ELBW, extremely low birthweight; EP, extremely preterm; HRQoL, health‐related quality of life; ICF, International Classification of Functioning, Disability and Health; LBW, low birthweight; MLP, moderate to late preterm; VLBW, very low birthweight; VP, very preterm.

^a^ Good quality (Newcastle–Ottawa Scale: 3 or 4 stars for cohort studies / 4 or 5 stars for cross‐sectional studies in the selection domain, AND 1 or 2 stars in the comparability domain, AND 2 or 3 stars in the outcome domain).

^b^ Fair quality (Newcastle–Ottawa Scale: 2 stars for cohort studies / 2 or 3 stars for cross‐sectional studies in the selection domain, AND 1 or 2 stars in the comparability domain, AND 2 or 3 stars in the outcome domain).

^c^ Poor quality (Newcastle–Ottawa Scale: 0 or 1 stars in the selection domain, OR 0 stars in the comparability domain, OR 0 or 1 stars in the outcome domain).

Data extracted from the articles included the number of individuals studied, the measure of motor skills, age at motor skills assessment, associated outcome, age at outcome assessment, measure of the association, mean or mean differences (as appropriate), and size of association. The tables were further arranged according to gestational age or birthweight groups: born extremely preterm or with extremely low birthweight (ELBW), born very preterm or with VLBW, and born moderate to late preterm or with low birthweight (LBW).

The size of the association, reported by the Pearson's correlation coefficient (*r*), the Spearman's rank correlation coefficient (*r*_*s*_), standardized beta (*β*), or difference in SD units between comparison groups, were interpreted as small (0.2), medium (0.5), or large (0.8).^27^ An odds ratio (OR) of 1.5 was considered as a small effect, an OR of 2 as a medium effect, and an OR of 3 as a large effect.^27^

## RESULTS

### Selection of articles for inclusion

A total of 5247 records were identified after searching the selected databases. After removing duplicates, 3593 unique records remained. Of these, 3375 were excluded and 218 full‐text articles were assessed. After full‐text assessment, 36 articles were eligible for inclusion and data extraction. An overview of the selection process is provided in Figure S1.

### Background characteristics

The 36 articles included were published between 1988 and 2025. Of these, 32 were cohort studies and four were cross‐sectional studies of individuals born preterm between 1977 and 2016. The articles originated from Australia (*n* = 8), Brazil (*n* = 3), Canada (*n* = 3), Europe (*n* = 16), the USA (*n* = 5), and Taiwan (*n* = 1). The criteria for the group born preterm were being born extremely preterm or with ELBW in 12 articles, being born very preterm or with VLBW in 14 articles, and being born moderate to late preterm or with LBW in 10 articles. Most studies excluded children with CP (*n* = 14), major cerebral damage, identifiable abnormalities, severe neurological impairment, or moderate‐to‐severe CP (*n* = 4) in the design. Some studies had no evidence of CP (*n* = 1), presented separate results for children with CP (*n* = 6), carried out sensitivity analyses excluding children with CP (*n* = 2), adjusted for CP in the analyses (*n* = 2), or stated similar results when children with CP were excluded from the analyses (*n* = 1). In six (17%) of the articles, no statement about CP was made. In 27 (75%) articles, considerations or adjustments for personal or environmental factors were performed (Table S4).

Motor skills were assessed using the Movement Assessment Battery for Children, Second Edition (Movement ABC‐2), the Movement ABC or its precursor the Test of Motor Impairment (*n* = 20), and the Bayley Scales of Infant and Toddler Development, Third Edition (Bayley‐III) or the BSID (*n* = 7) in most articles, but also according to age at walking (*n* = 1), Adult Developmental Coordination Disorders/Dyspraxia Checklist (*n* = 1), Ages & Stages Questionnaire, Second Edition (*n* = 1), Bruininks–Oseretsky Test of Motor Proficiency (*n* = 2), Grooved Pegboard test (*n* = 1), motor coordination of the modified Touwen examination (*n* = 1), Neurological Sensory Motor Developmental Assessment (*n* = 1), and the motor coordination subtest of the Beery–Buktenica Developmental Test of Visual‐Motor Integration, Sixth Edition (*n* = 1). Two articles used more than one assessment tool for motor skills, including the Alberta Infant Motor Scale and the Peabody Developmental Motor Scales.

The original 36 articles examined 56 associations between motor skills and outcomes in seven ICF chapters of activities and participation in children and adults born across the range of preterm birth. Of these, 13 (23%) were associations in Chapter 1 (Learning and applying knowledge), 12 (21%) in Chapter 2 (General tasks and demands), eight (14%) in Chapter 3 (Communication), seven (13%) in Chapter 5 (Self‐care), five (9%) in Chapter 7 (Interpersonal interactions and relationships), five (9%) in Chapter 8 (Major life areas or community), and six (11%) in Chapter 9 (Community, social and civic life). Eight articles reported associations in two chapters, four articles with three chapters, and one article with four different chapters (Table S4). One article reported associations for individuals born extremely preterm or with ELBW, individuals born very preterm or with VLBW, and individuals born moderate to late preterm or with LBW separately. Four (11%) articles included adult participants.

### Quality of the studies included in the review

Quality scores ranged from 4 to 9, with a mean Newcastle–Ottawa Scale score of 7.2 (SD = 1.2) for cohort studies and 6.0 (SD = 2.2) for cross‐sectional studies (Table S3). All cohort studies were rated high on representativeness, demonstration that the outcome was not present at the start of the study, and that enough follow‐up time for outcomes occurred. Ascertainment of motor skills, comparability with respect to confounders, and outcome assessment varied the most. Twenty‐three (64%) articles were rated as being of good quality, five (14%) as being of fair quality, and eight (22%) as being of poor quality. One article had a score of less than 5, indicating a high risk of bias.

### Associations between motor skills and learning and applying knowledge

In both children born extremely preterm or with ELBW and children born very preterm or with VLBW, abilities in reading, spelling, or mathematics were poorer in the group with motor difficulties compared with the group without motor difficulties at 8 to 9 years,^28^, ^29^, ^30^, ^31^ with effect sizes ranging from *−*0.63 to *−*1.0 (Table 1).^28^, ^30^

In children born extremely preterm or with ELBW, motor difficulties at 24 months were associated with higher attention‐deficit/hyperactivity scores at 7 to 9 years,^32^ and at 5 years to 6.5 years with poorer attention and executive function scores,^33^ as well as higher odds of having attention problems^34^ (Table 1). One large study did not report any association between Bayley‐III motor scores and attention‐deficit/hyperactivity problems at 22 to 26 months^35^ (Table 3), while another reported that motor difficulties in childhood were associated with attention in adults (22–36 years) but not at 8 years^36^ (Table 4).

In children born very preterm or with VLBW, Movement ABC‐2 Aiming & Catching and Manual Dexterity scores at 7 years, but not Balance scores, were associated with selective and sustained attention at 13 years. These associations were not found in the control group born at term^37^ (Table 4).

In children born moderate to late preterm or with LBW, there was a partial mediation effect of Grooved Pegboard test motor speed and motor coordination scores on the relationship between birthweight and inattention or hyperactivity^38^ (Table 4). However, Bayley‐III motor scores at 24 months did not correlate with attention problems,^39^ and Movement ABC‐2 scores at 5 years did not mediate the relationship between preterm birth and mathematical abilities^40^ (Table 4).

### Associations between motor skills and general tasks and demands

In children born extremely preterm or with ELBW, Bayley‐III motor scores at 22 to 26 months were associated with internalizing and externalizing problems^36^ (Table 3). Having motor difficulties was weakly to moderately associated with higher odds of total, internalizing, and externalizing problems at 6.5 years,^34^ and behavioural and externalizing problems at 8 to 9 years, corresponding to differences of 0.32 to 0.79 SD units (Table 1).^28^ At 11 to 13 years, Movement ABC scores were weakly associated with behavioural scores, but this association was similar in participants born with ELBW and participants born at term^41^ (Table 4).

In children born very preterm or with VLBW, motor difficulties were not associated with total difficulties on the Strength and Difficulties Questionnaire at 4 to 5 years,^42^ or deficient behaviour at 8 to 10 years^43^ (Table 1). Also in adults with VLBW, scores on the BSID Psychomotor Development Index (PDI) at 1 year, the Peabody Developmental Motor Scales at 5 years, or the Movement ABC at 14 years were not associated with adaptive and global functioning at 26 years^44^ (Table 3).

In children born moderate to late preterm or with LBW, BSID PDI scores at 24 months were moderately associated with the infant's affective and behavioural interactive style at 8 months, which was not seen in children born at term^45^ (Table 4). Bayley‐III motor scores were weakly associated with general competence in infancy, but not at toddler age;^46^ and Movement ABC scores at 6 years were weakly associated with behaviour reported by teachers but not by parents^47^ (Table 3). Age at walking was not associated with higher odds of poor problem‐solving at 4 years,^48^ and Bayley‐III motor scores at 24 months did not correlate with internalizing or externalizing behaviour at 6 years^39^ (Table 4).

### Associations between motor skills and communication

In children born extremely preterm, very preterm, and moderately preterm at 2 years, motor difficulties were associated with poor lexicon size^49^ (Table 1). In children born extremely preterm or with ELBW, Bayley‐III motor scores at 12 months were weakly to moderately associated with communicative gestures^50^ (Table 4), while motor coordination problems were associated with language scores at 5 years, corresponding to a difference of 0.59SD units^33^ (Table 1).

In children born very preterm or with VLBW, Bayley‐III motor scores at 8 months and 12 months were moderately associated with language scores at 3 years^51^ (Table 3). However, BSID PDI scores at 12 months, 18 months, and 24 months were not associated with communication scores^52^ (Table 3).

In children born moderate to late preterm or with LBW, there was no association between age at walking and poor communication scores at 4 years^48^ (Table 4).

### Associations between motor skills and self‐care

In children born extremely preterm or with ELBW, poorer Movement ABC scores at 5 years were strongly associated with less vigorous physical activity, but not with less leisure time physical activity at 11 years^53^ (Table 2).

In children born very preterm or with VLBW, Alberta Infant Motor Scale scores at 12 months, but not BSID PDI scores at 12 months, 18 months, and 24 months, were associated with daily living skills at 5 years^52^ (Table 3). Motor difficulties were associated with lower activities scores at 4 to 5 years, corresponding to a difference of −0.55SD units,^42^ while functional skills or caregiver assistance did not differ according to motor difficulties at 4 years^54^ (Table 2). Movement ABC‐2 Aiming & Catching scores, but not the other subscores, were associated with hours of structured physical activity assessed using accelerometers at 4 to 5 years, but not with other physical activity measures^55^ (Table 3). Motor competence was associated with moderate to vigorous physical activity at 12 to 20 years^56^ (Table 2).

In children born moderate to late preterm or with LBW, age at walking was weakly associated with poor personal and social skills at 4 years^48^ (Table 4).

### Associations between motor skills and interpersonal interaction and relationships

In children born extremely preterm or with ELBW, having motor difficulties was not associated with peer relations or social skills at 6.5 years^34^ (Table 2).

In children born very preterm or with VLBW, BSID PDI scores at 24 months, but not at 12 months and 18 months, were associated with socialization at 5 years^52^ (Table 3). Having motor difficulties at 4 to 5 years was associated with lower social functioning, corresponding to a difference of −0.49SD units^42^ (Table 2). However, peer problems did not differ^42^ and Movement ABC‐2 total score centiles were not associated with social and emotional loneliness at 11 years^57^ (Table 2). Still, Test of Motor Impairment scores at 6 years mediated the effect of birthweight on decreasing social self‐concept from 6 to 26 years^58^ (Table 4).

In infants and toddlers born moderate to late preterm or with LBW, Bayley‐III fine and gross motor scores were not associated with social persistence with adults^46^ (Table 3).

### Associations between motor skills and major life areas

In children born extremely preterm or with ELBW, being at risk for or having motor difficulties at 5 years were associated with twice the odds for school difficulties at 18 years, although this was not significant after adjustment for covariates^59^ (Table 2).

In children born very preterm or with VLBW, having motor difficulties at 3.5 years was strongly associated with the odds of receiving educational provision at 5.5 years,^60^ and 92% of children with motor difficulties at 6 years had school difficulties at 8 years^61^ (Table 2). Also, having motor difficulties was associated with literacy and numeracy underachievement and remedial education part‐time special education at 8 years.^29^ At 8 to 10 years, prevalence of inferior academic performance and not being able to follow school activities at the same level as others were almost twice as high in children with motor difficulties compared with children without motor difficulties, although this was not statistically significant^43^ (Table 2).

### Associations between motor skills and community, social and civic life

In children born extremely preterm or with ELBW, Movement ABC scores at 5 years were not associated with participation in organized sports activities at 11 years^53^ (Table 2).

In children born very preterm or with VLBW, a higher proportion of children with motor difficulties at 8 years did not participate in any sporting activity after school hours^29^ (Table 2). In a large study from 11 European countries, being at risk for or having motor difficulties were associated with lower physical and psychosocial health‐related quality of life (HRQoL) at 5 years.^62^ Similarly, motor difficulties were associated with lower HRQoL at 11 years.^63^ Lower scores on the Peabody Developmental Motor Scales at 5 years and the Movement ABC at 14 years, but not on the BSID PDI at 1 year, were weakly to moderately associated with lower physical and mental HRQoL at 28 years^44^ (Table 3). In another study, physical and psychosocial HRQoL did not differ between children with or without motor difficulties at 4 to 5 years^42^ (Table 2).

### Summary of results

Table 5 shows a summary of the main findings across ICF chapters and degree of preterm birth (*n* = 56). In children born extremely preterm or with ELBW, 13 (62%) associations between motor skills and other activities and participation outcomes were positive, four (19%) were inconsistent, and four (19%) indicated no associations. Fourteen (67%) of these associations were reported in articles of good quality, two (10%) in articles of fair quality, and five (24%) in articles of poor quality.

In children born very preterm or with VLBW, 11 (48%) associations were positive, four (17%) were inconsistent, and eight (35%) indicated no associations. Of these, 14 (61%) were reported in articles of good quality, six (26%) in articles of fair quality, and three (13%) in articles of poor quality.

In children born moderate to late preterm or with LBW, four (33%) associations were positive, two (17%) were inconsistent, and six (50%) indicated no associations. Of these, seven (58%) were reported in articles of good quality, one (8%) in an article of fair quality, and four (33%) in articles of poor quality.

## DISCUSSION

### Main findings

We identified 36 articles fulfilling the eligibility criteria, examining 56 associations between motor skills and outcomes of activities and participation in seven ICF chapters in children and adults born across the range of preterm birth. Most articles (89%) were from high‐income countries and most (89%) were cohort studies. The quality ranged from 4 to 9 on the Newcastle–Ottawa Scale, with approximately two‐thirds of the articles rated as being of good quality; only one article had a score of less than 5, indicating a high risk of bias.^26^ Motor skills were measured using the Movement ABC‐2, Movement ABC, or its precursor the Test of Motor Impairment in more than half of the articles. Associations of motor skills were reported with all chapters within the ICF Activities and Participation component, except Chapter 6 (Domestic life). The two most researched areas were Chapter 1 (Learning and applying knowledge) and Chapter 2 (General tasks and demands). Half of the associations examined showed that better motor skills were associated with better activities and participation outcomes. In contrast, 18% of the associations were inconsistent, and 32% showed no associations between motor skills and other outcomes in children and adults born extremely preterm or with ELBW, very preterm or with ELBW, and moderate to late preterm or with LBW. Effect sizes varied from small to large.

### Strengths and limitations

This is the first systematic review to assess the associations between different domains within the Activities and Participation component of the ICF in children and adults born preterm without CP. Strengths include the comprehensive systematic literature search, the use of two independent assessors, and the quality assessment of the articles included. Moreover, we included children and adults born across the range of preterm birth, from children born extremely preterm or with ELBW to children born moderate to late preterm or with LBW, and encompassing all ages, from infancy to adulthood.

Limitations include articles with varying study quality, sample sizes, assessment tools, and considerations regarding children with CP. Even though the Newcastle–Ottawa Scale is one of the most used scales for assessing quality and risk of bias in observational studies, it is not validated for cross‐sectional studies and the agreement between two assessors may be poor.^26^ However, most of the studies included in our systematic review were cohort studies, and disagreement between two independent assessors was solved using discussion. Like other studies using the Newcastle–Ottawa Scale, we adapted the scale for cross‐sectional studies and converted the scores for both cohort and cross‐sectional studies to Agency for Healthcare Research and Quality standards.^25^ To be rated as high quality, most criteria in all three domains of selection, comparability, and outcome must be fulfilled. Moreover, this is in line with the JBI critical appraisal checklists for cohort and analytical cross‐sectional studies.^19^ The quality scores were distributed across findings of associations between motor skills and the various ICF chapters and degree of preterm birth. This indicates that the results of this systematic review were not biased towards poorer study quality in some outcomes or gestational age or group birthweight.

The sample size ranged from only 24 individuals born preterm in one association analysis^44^ to 1074 in another.^36^ This may have resulted in a different power to detect associations because some small effects may be statistically significant only in large samples. Also, because there was substantial variation in the assessment tools used for both motor skills and the other outcomes of activities and participation, some null findings may reflect measurement insensitivity rather than the absence of association. Some articles (*n* = 9; 25%) examined associations in a control group born at term, strengthening the evidence that children and adults born preterm are particularly susceptible to co‐occurring adverse health outcomes. As almost 90% of the articles included came from high‐income countries, our findings may be less generalizable to low‐ and middle‐income countries.

We decided a priori to exclude participants with CP because this diagnosis is associated with limitations in activity and participation.^64^ However, as not all articles made explicit statements about CP, we cannot rule out that some studies included individuals with CP. However, more than 80% of the studies stated that individuals with CP were excluded in the design, presented separate results for participants with CP, performed sensitivity analyses excluding individuals with CP, or adjusted the analyses for CP.

### Use of the ICF framework in populations born preterm

We previously used the ICF framework in a systematic overview of a broad range of health outcomes and associations between outcomes from birth to adulthood in our own cohort of individuals born with VLBW.^7^ Despite the co‐occurrence of several difficulties in children and adults born preterm, no other systematic reviews have examined associations between motor skills and other outcomes of activities and participation within the ICF framework in this population.

Other articles using the ICF framework in populations born preterm include one systematic review^2^ and a few original articles.^3^, ^4^, ^5^, ^6^ The systematic review by FitzGerald et al.^2^ classified motor outcomes in children born very preterm aged 3 to 6 years according to body functions and structures, activity, and participation using the ICF for Children and Youth (ICF‐CY),^2^ whereas the original articles did not specifically address motor skills. Nevertheless, Sullivan et al.^3^ found the ICF model useful to better understand functional and participatory outcomes in adolescents and young adults with an array of biomedical and social risks, while Sell et al.^4^ studied the association between participation outcomes and body functions and structures in a German population‐based cohort. Moreover, a study by Giovannetti et al.^5^ using the ICF‐CY found that traditional assessment tools were poorly suited to evaluate the interaction between a person's functioning and environmental factors. Lastly, a qualitative study described the impacts of health conditions, environmental factors, and personal factors on the activities and participation of children born very preterm during middle childhood.^6^

We found the ICF to be a useful tool for classifying how motor skills relate to other outcomes of activities and participation in individuals born preterm. In this review, motor skills were conceptualized as an interconnected component of a broader developmental system, wherein development across different domains is mutually influential. We found motor skills to be associated with all ICF chapters within the activities and participation component, except for Chapter 6 (Domestic life). This might be because most studies involved children, while domestic activities, such as acquiring a place to live, housekeeping, and grocery shopping are taken care of by parents during childhood.

However, the ICF framework has received some criticism. Relevant to this systematic review is the lack of a clear conceptual and operational differentiation between activities and participation.^65^ Also, we categorized HRQoL in Chapter 9 (Community, social and civic life), but this concept is not explicitly incorporated into the ICF framework.^65^ While the ICF‐CY was developed to provide a better description of growth and development,^66^ we used the original ICF as our sample also included adults. However, the domains used in this systematic review do not differ substantially between the ICF^1^ and the ICF‐CY.^66^

### Interplay between motor skills and activities and participation

Motor skills are integral to functional development and do not operate in isolation. By definition, they are purposeful actions essential for mastering tasks of everyday life and are typically categorized into fine motor skills, which involve coordination of small muscle groups, and gross motor skills, which engage larger muscle groups.^10^ Motor development is closely linked to broader domains of functioning.^67^ In typically developing children, motor experiences are intertwined with development in other domains, with learning emerging through dynamic interactions with the physical environment and the social world.^68^

Both fine and gross motor skills contribute significantly to children's participation in daily routines, educational activities, and recreational contexts such as play, sports, and leisure. In early childhood, better motor skills may enhance children's ability to engage in play, which in turn gives opportunities to interact with peers and supports the development of both verbal and non‐verbal communication skills.^67^ Fine motor skills involve object manipulation and are critical for self‐care routines (e.g. grooming, brushing teeth) and academic skills (e.g. writing, drawing, typing). Gross motor skills underpin locomotor activities involving walking, running, skipping, and jumping. In summary, motor skills provide functional opportunities to participate in common childhood activities, which have meaningful impacts on child behaviours.^67^

The associations found in our systematic review do not imply a causal relationship between motor skills and other outcomes of activities and participation. Nevertheless, knowledge of these associations helps us to understand the complex interplay of activities and participation. Given the high comorbidity in children and adults born preterm,^7^ our findings may not be surprising. The broad spectrum of problems across several chapters of activities and participation suggests a common aetiology.^69^ The brain is particularly vulnerable in children born preterm,^70^, ^71^, ^72^, ^73^ causing neurodevelopmental problems that persist into adulthood. Nevertheless, while motor skills are clearly related to factors within the body functions and structures component of the ICF, such as poor growth, brain pathology, and visual impairments,^7^, ^13^ associations with activities and participation may be less evident.

We did not specifically address the personal and environmental factors of the ICF in this systematic review. However, 75% of the articles included considered or adjusted for personal or environmental factors, such as sex and socioeconomic factors. According to the biopsychosocial model of health, these factors have an important role and may contribute to differentiate outcomes in individuals born preterm given the same risk factors. A recent comprehensive review suggested that social determinants of the child's home environment, such as parental education, have an additive effect on the perinatal risk on language, cognitive, and behavioural outcomes, but there was less evidence for the effect on motor outcomes.^74^ Moreover, the importance of social factors seemed to increase with age.^74^ Thus, it is important to be aware that personal and environmental factors could influence the interplay between activities and participation.

### Clinical implications

This systematic review sheds light on the importance of addressing activity and participation outcomes in individuals born preterm. Most research has been done within body functions and structures. Our own research group published an overview of 58 articles from the Norwegian University of Science and Technology Low Birth Weight Life cohort, where only 16% of published articles had a main outcome within the Activities and Participation component of the ICF.^7^ These findings align with a systematic review of developmental coordination disorder in the general population, where Magalhaes et al.^75^ reported that only 14% of the articles published in the field included activities and participation. The Adults Born Preterm International Collaboration has published recommendations for common core assessments in studies of adults born preterm, which also include lifestyle, physical activity, social relationships, economic independence, reproduction, and HRQoL.^76^ As activities and participation are highly relevant for daily life and well‐being, we encourage more studies addressing these domains in individuals born preterm, especially as they grow older. Additionally, because the studies included in this review were mainly from high‐income countries, future research also needs to address these outcomes in individuals born preterm in low‐ and middle‐income countries.

Insights into associations between motor skills and several domains of activities and participation can inform screening, follow‐up, and intervention planning in the population born preterm. As motor difficulties may be evident early in life, they may serve as a marker of later challenges in other domains. Several studies supported the predictive value of early motor assessments for later cognitive abilities^77^ and also for behavioural outcomes and HRQoL.^78^, ^79^ Thus, the results of this systematic review may bring attention to the importance of both early screening and continued support. Guidelines for the follow‐up of children born extremely preterm is recommended until the children start school.^80^, ^81^, ^82^ However, difficulties with activities and participation may not be apparent until later, when demands in school and leisure time increase. Professionals in health care and educational systems should be aware of this and ensure that appropriate interventions are in place throughout the lifespan to help reduce the impact of difficulties with activities and participation.^83^

Given the need for ongoing support, it is important to consider how targeted interventions can promote motor skills and participation. While many interventions have been developed to enhance motor outcomes in children born preterm during the first years of life, there is less research on motor skill interventions during school age.^84^ Motor skills may be enhanced through task‐specific training, which may improve opportunities for learning and participation in play and sports. In addition, adapting activities and environments may enable children to engage in motor as well as daily life and social activities, thereby increasing their participation. Thus, knowledge of these associations may be essential for optimizing outcomes in individuals born preterm.

### Conclusion

This systematic review shows that motor skills are associated with learning and applying knowledge, general tasks and demands, communication, self‐care, interpersonal interactions and relationships, major life areas or community, social and civic life in children and adults without CP who are born preterm. Awareness of these associations could guide assessments and interventions in the population born preterm, with motor skills potentially serving as an early marker of later developmental challenges.

## Supporting information


**Figure S1:** PRISMA flow diagram of article selection for review.

**Table S1:** Documentation of search strategies for the bibliographic databases.

**Table S2:** Mapping of outcomes according to the ICF two‐level classification of activities and participation.

**Table S3:** Newcastle–Ottawa criteria for the present systematic review and quality assessment of included cohort and cross‐sectional studies.

**Table S4:** Characteristics of the included articles reporting associations between motor skills and outcomes of activities and participation in alphabetical order.

## Data Availability

The data that supports the findings of this study are available in the main manuscript and supplementary material of this article.
