# Supplementary material for: Motor skills and outcomes of activities and participation in children and adults born preterm without cerebral palsy: A systematic review
Source: Dev Med Child Neurol. 2026 Mar 27;68(9):1207–27. doi: 10.1111/dmcn.70242 (PMC13436715; doi:10.1111/dmcn.70242)
Supplement: Supplementary file 1 [file DMCN-68-1207-s001.docx]

**Table S3.** Newcastle-Ottawa criteria for the present systematic review.

**NEWCASTLE - OTTAWA QUALITY ASSESSMENT SCALE FOR COHORT STUDIES**

Note: A study can be awarded a maximum of one star for each numbered item within the Selection and Outcome categories. A maximum of two stars can be given for Comparability.

**Selection**

1) Representativeness of the ***preterm*** cohort

A) Truly representative of the average ***preterm population without cerebral palsy (not a sub-selection such as just those with medical illness or only males)*** in the community*

B) Somewhat representative of the average ***preterm population without cerebral palsy*** in the	community*

C) Selected group of users, ***e.g. with medical illness or only males***

D) No description of the derivation of the cohort

2) Selection of the ***non-preterm*** cohort

A) Drawn from the same community as the preterm cohort*

B) Drawn from a different source

C) No comparison cohort or description of the derivation of the non-exposed cohort

3) Ascertainment of ***motor skills***

A) Independent blind assessment using validated motor test*

B) Validated motor test*

C) Validated questionnaire

D) Non-validated assessment tool or questionnaire or no description

4) Demonstration that outcome of interest was not present at start of study ***(activity/participation outcome was not known when the participants were recruited)***

A) Yes*

B) No

**Comparability**

1) Comparability of cohorts on the basis of the design or analysis

A) The study ***considers or adjusts for sex and/or socioeconomic status as potential confounders of the association between motor skills and activity/participation outcome****

B) The study ***considers or adjusts for* *other potential confounders****

C) Cohorts are not comparable on the basis of design or analysis

**Outcome**

1) Assessment of outcome ***of activity/participation***

A) Independent blind assessment using validated assessment tool*

B) Validated assessment tool or questionnaire*

C) Non-validated assessment tool or questionnaire

D) No description

2) Was follow-up long enough for outcomes to occur

A) Yes ***(one year of age)****

B) No

3) Adequacy of follow up of cohorts

A) Complete follow up - all subjects accounted for*

B) Subjects lost to follow up unlikely to introduce bias ***>*** ***80% follow up rate up to 2 years or > 60% follow up rate beyond 2 years of eligible participants****

C) Follow up rate ***below criteria described in B)***

D) No statement

**NEWCASTLE - OTTAWA QUALITY ASSESSMENT SCALE ADAPTED FOR CROSS-SECTIONAL STUDIES**

Note: A study can be awarded a maximum of one star for each numbered item within the Selection category. A maximum of two stars can be given for Comparability and three stars for Outcome.

**Selection**

1) Representativeness of the ***preterm*** sample

A) Truly representative of the ***preterm population without cerebral palsy*** (all subjects or random sampling)*

B) Somewhat representative of the average in the ***preterm*** ***population*** ***without cerebral palsy*** (non-random sampling)*

C) Selected group of users/convenience sample

D) No description of the sampling strategy

2) Sample size

A) Justified and satisfactory*

B) Not justified

3) Non-response rate

A) The response rate is satisfactory ***(≥80%)****

B) The response rate is unsatisfactory ***(<80%)***

C) No description

4) Ascertainment of ***motor skills***

A) Independent blind assessment using validated motor test**

B) Validated motor test*

C) Validated questionnaire

D) Non-validated questionnaire or no description

**Comparability**

1) Comparability of studies on basis of the design or analysis

A) The study ***considers or adjusts for sex and/or socioeconomic status as potential confounders of the association between motor skills and activity/participation outcome*** *

B) The study ***considers or adjusts for other potential confounders****

C) Studies are not comparable on the basis of design or analysis

**Outcome**

1) Assessment of the outcome ***of activity/participation***

A) Independent blind assessment using validated assessment tool**

B) Validated assessment tool or questionnaire*

C) Non-validated assessment tool or questionnaire

D) No description

2) Statistical test

A) The statistical test used to analyze the data is clearly described and appropriate*

B) The statistical test is not appropriate, not described or incomplete

**Quality assessment of included cohort studies.**

| Citation | Representa-tiveness of the preterm cohort | Selection of the non-preterm cohort | Ascertainment of motor skills | Demonstration that outcome was not present at start of study | Comparability of studies on basis of design or analysis | Assessment of activity/ participation outcome | Follow-up long enough for outcomes to occur | Adequacy of follow up | Overall quality score | AHRQ standard |
| --- | --- | --- | --- | --- | --- | --- | --- | --- | --- | --- |
| Aubert et al. 2023^1^ | A* | C | B* | A* | A** | B* | A* | B* | 8 | Good |
| Benassi et al. 2016^2^ | A* | A* | B* | A* | C | A* | A* | D | 6 | Poor |
| Bogičević & van Baar 2019^3^ | A* | A* | A* | A* | A** | A* | A* | B* | 9 | Good |
| Bolk et al. 2018^4^ | A* | B | B* | A* | A** | B* | A* | B* | 8 | Good |
| Brown et al. 2023^5^ | A* | B | B* | A* | A* | B* | A* | B* | 7 | Good |
| Charkaluk et al. 2019^6^ | A* | C | C | A* | A** | B* | A* | C | 6 | Fair |
| Danks et al. 2017^7^ | A* | B | A* | A* | A** | B* | A* | C | 7 | Good |
| Davis et al. 2007^8^ | A* | A* | A* | A* | A** | B* | A* | B* | 9 | Good |
| Engan et al. 2020^9^ | A* | B | B* | A* | A** | C | A* | B* | 7 | Good |
| FitzGerald et al. 2024^10^ | A* | C | A* | A* | B* | A* | A* | D | 6 | Good |
| Greenberg & Crnic 1988^11^ | A* | A* | B* | A* | C | C | A* | D | 5 | Poor |
| Hasler & Akshoomoff 2019^12^ | A* | A* | B* | A* | B* | B* | A* | D | 7 | Good |
| Helin et al. 2022^13^ | A* | C | B* | A* | A* | B* | A* | B* | 7 | Good |
| Holsti et al. 2002^14^ | A* | A* | B* | A* | C | B* | A* | B* | 7 | Poor |
| Howe et al. 2016^15^ | A* | C | B* | A* | A** | B* | A* | B* | 8 | Good |
| Jeyaseelan et al. 2006^16^ | B* | C | A* | A* | A** | B* | A* | B* | 8 | Good |
| Jongmans et al. 1997^17^ | A* | B | B* | A* | C | B* | A* | B* | 6 | Poor |
| Korkman et al. 2008^18^ | A* | C | B* | B | B* | B* | A* | B* | 6 | Poor |
| Liu et al. 2021^19^ | A* | A* | B* | A* | A* | B* | A* | C | 7 | Good |
| Lowe et al. 2019^20^ | A* | C | B* | A* | A** | B* | A* | B* | 8 | Good |
| Marlow et al. 1993^21^ | A* | B | B* | A* | A* | B* | A* | B* | 7 | Good |
| Martel et al. 2007^22^ | A* | A* | B* | A* | A** | B* | A* | B* | 9 | Good |
| Panceri et al. 2022^23^ | A* | C | B* | A* | B* | B* | A* | C | 6 | Good |
| Poole et al. 2015^24^ | A* | B | C | A* | B* | B* | A* | B* | 6 | Fair |
| Roberts et al. 2011^25^ | A* | A* | A* | A* | C | B* | A* | B* | 7 | Poor |
| Spittle et al. 2022^26^ | A* | C | A* | A* | A** | B* | A* | B* | 8 | Good |
| Uusitalo et al. 2020^27^ | A* | C | B* | A* | A** | B* | A* | B* | 8 | Good |
| van Dokkum et al. 2021^28^ | A* | A* | D | A* | A** | B* | A* | B* | 8 | Good |
| Verkerk et al. 2014^29^ | A* | B | B* | A* | B* | C | A* | B* | 6 | Fair |
| Wiingreen et al. 2024^30^ | A* | A* | B* | A* | A** | A* | A* | B* | 9 | Good |
| Wocadlo & Rieger 2008^31^ | A* | C | B* | A* | C | B* | A* | C | 5 | Poor |
| Wollum et al. 2022^32^ | A* | A* | A* | A* | A* | B* | A* | B* | 8 | Good |

**Quality assessment of included cross-sectional studies.**

| Citation | Representa-tiveness of the preterm sample | Sample size | Non-response rate | Ascertainment of motor skills | Comparability of studies on basis of design or analysis | Assessment of activity/participation outcome | Statistical test | Overall quality score | AHRQ standard |
| --- | --- | --- | --- | --- | --- | --- | --- | --- | --- |
| Blasco et al. 2023^33^ | A* | B | C | B* | C | B* | A* | 4 | Poor |
| Maggi et al. 2014^34^ | C | A* | C | B* | B* | B* | A* | 5 | Fair |
| Moreira et al. 2014^35^ | A* | B | A* | B* | A* | B* | A* | 6 | Fair |
| Proulx et al. 2017^36^ | A* | A* | B | A** | A** | A** | A* | 9 | Good |

Abbreviation: AHRQ, Agency for Healthcare Research and Quality

**Thresholds for converting the Newcastle-Ottawa scales to AHRQ standards (good, fair, and poor) for cohort studies^#^**

Good quality: 3 or 4 stars in selection domain AND 1 or 2 stars in comparability domain AND 2 or 3 stars in outcome domain

Fair quality: 2 stars in selection domain AND 1 or 2 stars in comparability domain AND 2 or 3 stars in outcome domain

Poor quality: 0 or 1 star in selection domain OR 0 stars in comparability domain OR 0 or 1 stars in outcome domain

**Thresholds for converting the Newcastle-Ottawa scales to AHRQ standards (good, fair, and poor) for cross-sectional studies^#^**

Good quality: 4 or 5 stars in selection domain AND 1 or 2 stars in comparability domain AND 2 or 3 stars in outcome domain

Fair quality: 2 or 3 stars in selection domain AND 1 or 2 stars in comparability domain AND 2 or 3 stars in outcome domain

Poor quality: 0 or 1 star in selection domain OR 0 stars in comparability domain OR 0 or 1 stars in outcome domain

**^#^Reference:** Kim H, Kim K, Kim YH. Associations between mental illness and cancer: a systematic review and meta-analysis of observational studies. Eur Rev Med Pharmacol Sci. 2022;26(14):4997–5007.

**References**

1. Aubert AM, Costa R, Johnson S, et al. Developmental motor problems and health-related quality of life in 5-year-old children born extremely preterm: A European cohort study. Dev Med Child Neurol. 2023;65(12):1617–1628.
2. Benassi E, Savini S, Iverson JM, et al. Early communicative behaviors and their relationship to motor skills in extremely preterm infants. Res Dev Disabil. 2016;48:132–144.
3. Bogičević L, Verhoeven M, van Baar AL. Toddler skills predict moderate-to-late preterm born children's cognition and behaviour at 6 years of age. PLoS One. 2019;14(11):e0223690.
4. Bolk J. FA, Hafström M., Åden U., Serenius F. Developmental Coordination Disorder and Its Association With Developmental Comorbidities at 6.5 Years in Apparently Healthy Children Born Extremely Preterm. JAMA pediatrics. 2018;172(8):765–774.
5. Brown RN, Burnett AC, Thompson DK, et al. Motor performance and attention outcomes in children born very preterm. Dev Med Child Neurol. 2023;65(11):1501–1510.
6. Charkaluk ML, Rousseau J, Benhammou V, et al. Association of Language Skills with Other Developmental Domains in Extremely Very and Moderately Preterm Children: EPIPAGE 2 Cohort Study. The Journal of pediatrics. 2019;208:114.
7. Danks M, Cherry K, Burns YR, et al. Are behaviour problems in extremely low-birthweight children related to their motor ability? Acta paediatrica (Oslo Norway : 1992). 2017;106(4):568–572.
8. Davis NM, Ford GW, Anderson PJ, et al. Developmental coordination disorder at 8 years of age in a regional cohort of extremely-low-birthweight or very preterm infants. Dev Med Child Neurol. 2007;49(5):325–330.
9. Engan M, Engeseth MS, Fevang S, et al. Predicting physical activity in a national cohort of children born extremely preterm. Early Hum Dev. 2020;145.
10. FitzGerald TL, Cameron KL, Albesher RA, et al. Associations between physical activity and development in preschool-aged children born <30 weeks' gestation: a cohort study. Arch Dis Child Fetal Neonatal Ed. 2024;109(6):602–608.
11. Greenberg MT, Crnic KA. Longitudinal predictors of developmental status and social interaction in premature and full-term infants at age two. Child Dev. 1988;59(3):554–570.
12. Hasler HM, Akshoomoff N. Mathematics ability and related skills in preschoolers born very preterm. Child Neuropsychol. 2019;25(2):162–178.
13. Helin M, Karukivi M, Haataja L, et al. Motor Performance in Association with Perceived Loneliness and Social Competence in 11-Year-Old Children Born Very Preterm. Children. 2022;9(5).
14. Holsti L, Grunau RVE, Whitfield MF. Developmental coordination disorder in extremely low birth weight children at nine years. J Dev Behav Pediatr. 2002;23(1):9–15.
15. Howe TH, Sheu CF, Hsu YW, et al. Predicting neurodevelopmental outcomes at preschool age for children with very low birth weight. Res Dev Disabil. 2016;48:231–241.
16. Jeyaseelan D, O'Callaghan M, Neulinger K, et al. The association between early minor motor difficulties in extreme low birth weight infants and school age attentional difficulties. Early Hum Dev. 2006;82(4):249–255.
17. Jongmans M, Mercuri E, De Vries L, et al. Minor neurological signs and perceptual-motor difficulties in prematurely born children. Arch Dis Child Fetal Neonatal Ed. 1997;76(1):F9–F14.
18. Korkman M, Mikkola K, Ritari N, et al. Neurocognitive test profiles of extremely low birth weight five-year-old children differ according to neuromotor status. Dev Neuropsychol. 2008;33(5):637–655.
19. Liu Y, Mendonça M, Bartmann P, et al. Very preterm birth and trajectories of domain-specific self-concept from childhood into adulthood. Dev Psychopathol. 2021:1–12.
20. Lowe JR, Fuller JF, Do BT, et al. Behavioral problems are associated with cognitive and language scores in toddlers born extremely preterm. Early Hum Dev. 2019;128:48–54.
21. Marlow N, Roberts L, Cooke R. Outcome at 8 years for children with birth weights of 1250 g or less. Arch Dis Child. 1993;68(3 SUPPL.):286–290.
22. Martel MM, Lucia VC, Nigg JT, et al. Sex differences in the pathway from low birth weight to inattention/hyperactivity. J Abnorm Child Psychol. 2007;35(1):87–96.
23. Panceri C, Silveira RC, Procianoy RS, et al. Motor development in the first year of life predicts impairments in cognition and language at 3 years old in a Brazilian preterm cohort of low-income families. Front Neurosci. 2022;16.
24. Poole KL, Schmidt LA, Missiuna C, et al. Motor coordination and mental health in extremely low birth weight survivors during the first four decades of life. Res Dev Disabil. 2015;43-44:87–96.
25. Roberts G, Anderson PJ, Davis N, et al. Developmental coordination disorder in geographic cohorts of 8-year-old children born extremely preterm or extremely low birthweight in the 1990s. Dev Med Child Neurol. 2011;53(1):55–60.
26. Spittle AJ, Olsen JE, FitzGerald TL, et al. School Readiness in Children Born <30 Weeks' Gestation at Risk for Developmental Coordination Disorder: A Prospective Cohort Study. J Dev Behav Pediatr. 2022;43(5):E312–E319.
27. Uusitalo K, Haataja L, Nyman A, et al. Preterm children's developmental coordination disorder cognition and quality of life: a prospective cohort study. BMJ Paediatrics Open. 2020;4(1):e000633.
28. van Dokkum NH, Reijneveld SA, Bos AF, et al. Attainment of smiling and walking in infancy associates with developmental delays at school entry in moderately-late preterm children: a community-based cohort study. BMC Pediatr. 2021;21(1):84.
29. Verkerk G, Jeukens-Visser M, van Wassenaer-Leemhuis A, et al. The relationship between multiple developmental difficulties in very low birth weight children at 3½ years of age and the need for learning support at 5 years of age. Res Dev Disabil. 2014;35(1):185–191.
30. Wiingreen R, Greisen G, Esbjorn BH, et al. Five-year neurodevelopmental assessment of extremely preterm or extremely low birthweight infants: Association with school performance. Acta Paediatr. 2025;114(1):131–140.
31. Wocadlo C, Rieger I. Motor impairment and low achievement in very preterm children at eight years of age. Early Hum Dev. 2008;84(11):769–776.
32. Wollum AEK, Berdal EK, Iversen JM, et al. Outcomes and predictors of functioning mental health and health-related quality of life in adults born with very low birth weight: a prospective longitudinal cohort study. BMC Pediatr. 2022;22(1):628.
33. Blasco P, Saxton S, Gullion LM, et al. Assessment of Mastery Motivation and Neurodevelopment of Young Children at High Risk for Developmental Delays. Journal of Intelligence. 2023;11(6).
34. Maggi EF, Magalhães LC, Campos AF, et al. Preterm children have unfavorable motor cognitive and functional performance when compared to term children of preschool age. J Pediatr (Rio J). 2014;90(4):377–383.
35. Moreira RS, Magalhães LC, Dourado JS, et al. Factors influencing the motor development of prematurely born school-aged children in Brazil. Res Dev Disabil. 2014;35(9):1941–1951.
36. Proulx K, Majnemer A, Dahan-Oliel N, et al. Factors Associated With Moderate to Vigorous Physical Activity in Adolescents Born Preterm. Pediatr Exerc Sci. 2017;29(2):260–267.
