# Supplementary material for: Motor skills and outcomes of activities and participation in children and adults born preterm without cerebral palsy: A systematic review
Source: Dev Med Child Neurol. 2026 Mar 27;68(9):1207–27. doi: 10.1111/dmcn.70242 (PMC13436715; doi:10.1111/dmcn.70242)
Supplement: Supplementary file 2 [file DMCN-68-1207-s002.docx]

**Table S2.** Classification of outcomes according to the two-level classification of activities and participation within the ICF.

| **Component** | **Activities and participation** | | | | | | | | |
| --- | --- | --- | --- | --- | --- | --- | --- | --- | --- |
| Chapter headings | Chapter 4  Mobility | Chapter 1  Learning and Applying Knowledge | Chapter 2 General tasks and demands | Chapter 3 Communication | Chapter 5 Self-care | Chapter 6 Domestic life | Chapter 7 Interpersonal interactions and relationships | Chapter 8  Major life areas | Chapter 9 Community, social and civic life |
| First level domains | Changing and maintaining body position (d410-d429)  Carrying, moving and handling objects (d430-d449)  Walking and moving  (d450-d469)  Moving around using transportation (d470-d489) | Purposeful sensory experiences (d110-d129)  Basic learning (d130-159)  Applying knowledge (d160-179) |  | Communicating - receiving (d310-d329)  Communicating - producing (d330-d349)  Conversation and use of communication devices and techniques (d350-  d369) |  | Acquisition of necessities (d610-d629)  Household tasks (d630-d649)  Caring for household objects and assisting others (d650-d669) | General inter-personal interactions (d710-d729)  Particular interpersonal relationships (d730-d779) | Education (d810-d839)  Work and employment (d840-d859)  Economic life (d860-d879) |  |
| Second level domains | d410 Changing basic body position  d415 Maintaining a body position  d420 Transferring oneself  d429 Changing and maintaining body position, other specified and unspecified  d430 Lifting and carrying objects  d435 Moving objects with lower extremities  d440 Fine hand use  d445 Hand and arm use  d449 Carrying, moving and handling objects, other specified and unspecified  Walking and moving (d450-d469)  d450 Walking  d455 Moving around  d460 Moving around in different locations  d465 Moving around using equipment  d469 Walking and moving, other specified and unspecified  d470 Using transportation  d475 Driving  d480 Riding animals for transportation  d489 Moving around using transportation, other specified and unspecified  d498 Mobility, other specified  d499 Mobility, unspecified | d110 Watching  d115 Listening  d120 Other purposeful sensing  d129 Purposeful sensory experiences, other specified and unspecifiedd130 Copying  d135 Rehearsing  d140 Learning to read  d145 Learning to write  d150 Learning to calculate  d155 Acquiring skills  d159 Basic learning, other specified and unspecified  Applying knowledge (d160-d179)  d160 Focusing attention  d163 Thinking  d166 Reading  d170 Writing  d172 Calculating  d175 Solving problems  d177 Making decisions  d179 Applying knowledge, other specified and unspecified  d198 Learning and applying knowledge, other specified  d199 Learning and applying knowledge, unspecified | d210 Undertaking a single task  d220 Undertaking multiple tasks  d230 Carrying out daily routine  d240 Handling stress and other psychological demands  d298 General tasks and demands, other specified  d299 General tasks and demands, unspecified | d310 Communicating with - receiving - spoken messages  d315 Communicating with - receiving - nonverbal messages  d320 Communicating with - receiving - formal sign language messages  d325 Communicating with - receiving - written messages  d329 Communicating - receiving, other specified and unspecified  d350 Conversation  d355 Discussion  d360 Using communication devices and techniques  d369 Conversation and use of communication devices and techniques, other  specified and unspecified  d398 Communication, other specified  d399 Communication, unspecified | d510 Washing oneself  d520 Caring for body parts  d530 Toileting  d540 Dressing  d550 Eating  d560 Drinking  d570 Looking after one’s health  d598 Self-care, other specified | d610 Acquiring a place to live  d620 Acquisition of goods and services  d629 Acquisition of necessities, other specified and unspecified  d630 Preparing meals  d640 Doing housework  d649 Household tasks, other specified and unspecified  d650 Caring for household objects  d660 Assisting others  d669 Caring for household objects and assisting others, other specified and  unspecified  d698 Domestic life, other specified  d699 Domestic life, unspecified | d710 Basic interpersonal interactions  d720 Complex interpersonal interactions  d729 General interpersonal interactions, other specified and unspecified  d730 Relating with strangers  d740 Formal relationships  d750 Informal social relationships  d760 Family relationships  d770 Intimate relationships  d779 Particular interpersonal relationships, other specified and unspecified  d798 Interpersonal interactions and relationships, other specified  d799 Interpersonal interactions and relationships, unspecified | d810 Informal education  d815 Preschool education  d820 School education  d825 Vocational training  d830 Higher education  d839 Education, other specified and unspecified  d840 Apprenticeship (work preparation)  d845 Acquiring, keeping and terminating a job  d850 Remunerative employment  d855 Non-remunerative employment  d859 Work and employment, other specified and unspecified  d860 Basic economic transactions d865 Complex economic transactions d870 Economic self-sufficiency d879 Economic life, other specified and unspecified d898 Major life areas, other specified d899 Major life areas, unspecified | d910 Community life  d920 Recreation and leisure  d930 Religion and spirituality  d940 Human rights  d950 Political life and citizenship  d998 Community, social and civic life, other specified  d999 Community, social and civic life, unspecified |
| Own categoriza-tion | Motor skills | Attention  Academic skills | Behavior  General tasks | Communication | Daily living  Physical activity |  | Socialization | Education | Activity participation  HRQoL |
| Examples of inclusions | Motor skills (MABC, AIMS, ASQ, BSID-III, BOTMP)  Motor problems/difficulties  Balance  Fine motor skills  Gross motor skills  Fine motor coordination  Gross motor coordination  Walking  Motor coordination | Attention skills, Attention problems  Focusing attention  Learning difficulties  Problem solving (ASQ)  Reading (WRAT)  Spelling (WRAT)  Mathematic ability (WRAT) | Executive function in daily life (BRIEF)  Behavior (CBCL)  General development and functioning (GAF)  Personal/social (ASQ) | Language skills (BSID-III)  Communication (ASQ) | Functional skills (PEDI)  Caregiver Assistance (PEDI) Physical activity | Personal/social (ASQ) | Parent-child/Child-parent relationships (d7600/01)  Friends  Bullying | Academic performance | Participation  Leisure time activities  Activity level  Quality of life |
| Examples of exclusions | Minor neurological dysfunction  Visual motor integration (VMI)  General movements (GMA)  Fidgety movements (GMA)  Diagnoses (DCD, F82) | Vision  Sensory processing  Processing speed  Diagnoses (ADHD)  Intellectual functions (b117)  Attention functions (b140)  Memory functions b144)  Emotional functions (b152)  Perceptual functions (b156)  Visual perception (b1561)  Perception |  |  |  |  | Diagnoses (ASD) |  |  |

Abbreviations: ADHD, Attention Deficit Hyperactivity Disorder; AIMS, Alberta Infant Motor Scale; ASD, Autism Spectrum Disorder; ASQ, Ages & Stages Questionnaire; BOTMP, Bruininks-Oseretsky Test of Motor Proficiency; BRIEF, Behavior Rating Inventory of Executive Function; BSID-III, Bayley Scales of Infant and Toddler Development – Third Edition; CBCL, Child Behavior Checklist; GAF, Global Assessment of Functioning; GMA, General Movements Assessment; HRQoL, Health-related quality of life; ICF, International Classification of Functioning, Disability and Health; Movement ABC, Movement Assessment Battery for Children; PEDI, Pediatric Evaluation of Disability Inventory; VMI, Beery-Buktenica Developmental Test of Visual-Motor Integration; WRAT, Wide Range Achievement Test.
