# Supplementary material for: Motor skills and outcomes of activities and participation in children and adults born preterm without cerebral palsy: A systematic review
Source: Dev Med Child Neurol. 2026 Mar 27;68(9):1207–27. doi: 10.1111/dmcn.70242 (PMC13436715; doi:10.1111/dmcn.70242)
Supplement: Supplementary file 3 [file DMCN-68-1207-s003.docx]

**Table S1.** Documentation of search strategies for the bibliographic databases.

**MEDLINE via EBSCOhost**

| Row | Search query |
| --- | --- |
| S1 | (MH "Infant, Low Birth Weight+") |
| S2 | (MH "Infant, Very Low Birth Weight+") |
| S3 | (MH "Infant, Extremely Low Birth Weight") |
| S4 | (MH "Infant, Small for Gestational Age") |
| S5 | TI "low birth weight*" OR AB "low birth weight*" OR SU "low birth weight*" |
| S6 | TI "low weight at birth" OR AB "low weight at birth" OR SU "low weight at birth" |
| S7 | TI small N2 "gestational age" OR AB small N2 "gestational age" OR SU small N2 "gestational age" |
| S8 | TI lbw OR AB lbw OR SU lbw |
| S9 | TI vlbw OR AB vlbw OR SU vlbw |
| S10 | TI elbw OR AB elbw OR SU elbw |
| S11 | TI sfga OR AB sfga OR SU sfga |
| S12 | (MH "Premature Birth") |
| S13 | (MH "Infant, Premature+") |
| S14 | (MH "Infant, Extremely Premature") |
| S15 | TI (prematur* OR 'pre matur*' OR preterm* OR 'pre term*') N1 (birth* OR deliver* OR baby OR babies OR neonat* OR 'new born*' OR deliver* OR infant* OR child* OR very OR extrem*) OR AB (prematur* OR 'pre matur*' OR preterm* OR 'pre term*') N1 (birth* OR deliver* OR baby OR babies OR neonat* OR 'new born*' OR deliver* OR infant* OR child* OR very OR extrem*) OR SU (prematur* OR 'pre matur*' OR preterm* OR 'pre term*') N1 (birth* OR deliver* OR baby OR babies OR neonat* OR 'new born*' OR deliver* OR infant* OR child* OR very OR extrem*) |
| S16 | S1 OR S2 OR S3 OR S4 OR S5 OR S6 OR S7 OR S8 OR S9 OR S10 OR S11 OR S12 OR S13 OR S14 OR S15 |
| S17 | (MH "Motor Skills") |
| S18 | (MH "Motor Skills Disorders") |
| S19 | TI (motor* OR movement) N0 (abilit* OR capabilit* OR compet* OR function* OR speed OR skill* OR coordinat* OR 'co ordinat*' OR fine OR gross OR disability* OR disfunction* OR dysfunction* OR difficult* OR disorder* OR disturbance* OR delay* OR deficit* OR impairment* OR problem* OR challeng* OR clums* OR effect* OR predictor*) OR AB (motor* OR movement) N0 (abilit* OR capabilit* OR compet* OR function* OR speed OR skill* OR coordinat* OR 'co ordinat*' OR fine OR gross OR disability* OR disfunction* OR dysfunction* OR difficult* OR disorder* OR disturbance* OR delay* OR deficit* OR impairment* OR problem* OR challeng* OR clums* OR effect* OR predictor*) OR SU (motor* OR movement) N0 (abilit* OR capabilit* OR compet* OR function* OR speed OR skill* OR coordinat* OR 'co ordinat*' OR fine OR gross OR disability* OR disfunction* OR dysfunction* OR difficult* OR disorder* OR disturbance* OR delay* OR deficit* OR impairment* OR problem* OR challeng* OR clums* OR effect* OR predictor*) |
| S20 | TI (coordinat* OR 'co ordinat*' OR balance) N0 (abilit* OR capabilit* OR compet* OR function* OR speed OR skill* OR fine OR gross OR disability* OR disfunction* OR dysfunction* OR difficult* OR disorder* OR disturbance* OR delay* OR deficit* OR impairment* OR problem* OR challeng* OR clums*) OR AB (coordinat* OR 'co ordinat*' OR balance) N0 (abilit* OR capabilit* OR compet* OR function* OR speed OR skill* OR fine OR gross OR disability* OR disfunction* OR dysfunction* OR difficult* OR disorder* OR disturbance* OR delay* OR deficit* OR impairment* OR problem* OR challeng* OR clums*) OR SU (coordinat* OR 'co ordinat*' OR balance) N0 (abilit* OR capabilit* OR compet* OR function* OR speed OR skill* OR fine OR gross OR disability* OR disfunction* OR dysfunction* OR difficult* OR disorder* OR disturbance* OR delay* OR deficit* OR impairment* OR problem* OR challeng* OR clums*) |
| S21 | (MH "Apraxias+") |
| S22 | TI dyspraxia OR AB dyspraxia OR SU dyspraxia |
| S23 | TI apraxia OR AB apraxia OR SU apraxia |
| S24 | S17 OR S18 OR S19 OR S20 OR S21 OR S22 OR S23 |
| S25 | MH ("Epidemiologic Studies") OR (TI "case control" OR AB "case control") OR (TI "case-control" OR AB "case-control") OR (TI case OR AB case) OR (TI cases OR AB cases) OR (TI control OR AB control) OR (TI controls OR AB controls) OR (TI "cohort study" OR AB "cohort study") OR (TI "cohort analysis" OR AB "cohort analysis") OR (TI "follow up study" OR AB "follow up study") OR (TI "observational study" OR AB "observational study") OR (TI longitudinal OR AB longitudinal) OR (TI retrospective OR AB retrospective) OR (TI cross sectional OR AB cross sectional) OR (TI questionnaire OR AB questionnaire) OR (TI questionnaires OR AB questionnaires) OR (TI survey OR AB survey) |
| S26 | S16 AND S24 AND S25 |
| S27 | MH ("Animals") NOT MH ("Humans") |
| S28 | PT Letter OR PT Editorial |
| S29 | S26 NOT (S27 OR S28) |

**Embase via Embase.com**

| Row | Search query |
| --- | --- |
| #1 | 'low birth weight'/de |
| #2 | 'small for gestational age'/de |
| #3 | 'very low birth weight'/de |
| #4 | 'extremely low birth weight'/de |
| #5 | 'low birth weight*':ti,ab,kw |
| #6 | 'low weight at birth':ti,ab,kw |
| #7 | (small NEAR/3 'gestational age'):ti,ab,kw |
| #8 | lbw:ti,ab,kw |
| #9 | vlbw:ti,ab,kw |
| #10 | elbw:ti,ab,kw |
| #11 | sfga:ti,ab,kw |
| #12 | sga:ti,ab,kw |
| #13 | 'prematurity'/de |
| #14 | 'very premature birth'/de |
| #15 | 'extremely premature birth'/de |
| #16 | ((prematur* OR 'pre matur*' OR preterm* OR 'pre term*') NEAR/2 (birth* OR deliver* OR baby OR babies OR neonat* OR 'new born*' OR deliver* OR infant* OR child* OR very OR extrem*)):ti,ab,kw |
| #17 | #1 OR #2 OR #3 OR #4 OR #5 OR #6 OR #7 OR #8 OR #9 OR #10 OR #11 OR #12 OR #13 OR #14 OR #15 OR #16 |
| #18 | 'motor performance'/de |
| #19 | 'motor dysfunction'/de |
| #20 | 'motor dysfunction assessment'/de |
| #21 | ((motor* OR movement) NEAR/1 (abilit* OR capabilit* OR compet* OR function* OR speed OR skill* OR coordinat* OR 'co ordinat*' OR fine OR gross OR disability* OR disfunction* OR dysfunction* OR difficult* OR disorder* OR disturbance* OR delay* OR deficit* OR impairment* OR problem* OR challeng* OR clums* OR effect* OR predictor*)):ti,ab,kw |
| #22 | ((coordinat* OR 'co ordinat*' OR balance) NEAR/1 (abilit* OR capabilit* OR compet* OR function* OR speed OR skill* OR fine OR gross OR disability* OR disfunction* OR dysfunction* OR difficult* OR disorder* OR disturbance* OR delay* OR deficit* OR impairment* OR problem* OR challeng* OR clums*)):ti,ab,kw |
| #23 | 'developmental coordination disorder'/de |
| #24 | dyspraxia:ti,ab,kw |
| #25 | apraxia:ti,ab,kw |
| #26 | #18 OR #19 OR #20 OR #21 OR #22 OR #23 OR #24 OR #25 |
| #27 | 'clinical study'/exp OR 'cohort analysis'/exp OR 'case control':ab,ti OR 'case-control':ab,ti OR ((case:ab,ti OR cases:ab,ti) AND (control:ab,ti OR controls:ab,ti)) OR 'cohort study':ab,ti OR 'cohort analysis':ab,ti OR 'follow up study':ab,ti OR 'follow-up study':ab,ti OR 'observational study':ab,ti OR longitudinal:ab,ti OR retrospective:ab,ti OR 'cross sectional':ab,ti OR questionnaire:ab,ti OR questionnaires:ab,ti OR survey:ab,ti OR 'epidemiological study':ab,ti |
| #28 | #17 AND #26 AND #27 |
| #29 | 'animal'/de NOT 'human'/de |
| #30 | 'editorial':it OR 'letter':it OR 'abstract':it |
| #31 | #28 NOT (#29 OR #30) |

**Web of Science Core Collection**

| Row | Search Query |
| --- | --- |
| 1 | "low birth weight*" (Topic) |
| 2 | "low weight at birth" (Topic) |
| 3 | small NEAR/2 "gestational age" (Topic) |
| 4 | "lbw" (Topic) |
| 5 | "vlbw" (Topic) |
| 6 | "elbw" (Topic) |
| 7 | "sfga" (Topic) |
| 8 | "sga" (Topic) |
| 9 | TS=((prematur* OR "pre matur*" OR preterm* OR "pre term*") NEAR/1 (birth* OR deliver* OR baby OR babies OR neonat* OR "new born*" OR deliver* OR infant* OR child* OR very OR extrem*)) |
| 10 | #9 OR #8 OR #7 OR #6 OR #5 OR #4 OR #3 OR #2 OR #1 |
| 11 | TS=((motor* OR movement) NEAR/0 (abilit* OR capabilit* OR compet* OR function* OR speed OR skill* OR coordinat* OR "co ordinat*" OR fine OR gross OR disability* OR disfunction* OR dysfunction* OR difficult* OR disorder* OR disturbance* OR delay* OR deficit* OR impairment* OR problem* OR challeng* OR clums* OR effect* OR predictor*)) |
| 12 | TS=((coordinat* OR "co ordinat*" OR balance) NEAR/0 (abilit* OR capabilit* OR compet* OR function* OR speed OR skill* OR fine OR gross OR disability* OR disfunction* OR dysfunction* OR difficult* OR disorder* OR disturbance* OR delay* OR deficit* OR impairment* OR problem* OR challeng* OR clums*)) |
| 13 | "dyspraxia" (Topic) |
| 14 | "apraxia" (Topic) |
| 15 | #14 OR #13 OR #12 OR #11 |
| 16 | TI=("case control" OR "case-control" OR ((case OR cases) AND (control OR controls)) OR "cohort study" OR "cohort analysis" OR "follow up study" OR "follow-up study" OR "observational study" OR longitudinal" OR retrospective OR "cross sectional" OR questionnaire" OR questionnaires OR survey OR "epidemiological study") OR AB=("case control" OR "case-control" OR ((case OR cases) AND (control OR controls)) OR "cohort study" OR "cohort analysis" OR "follow up study" OR "follow-up study" OR "observational study" OR longitudinal" OR retrospective OR "cross sectional" OR questionnaire" OR questionnaires OR survey OR "epidemiological study") |
| 17 | #10 AND #15 AND #16 |
| 18 | DT=(Editorial Material) OR DT=(Letter) OR DT=(Meeting Abstract) |
| 19 | #17 NOT #18 |
