# Supplementary material for: Motor skills and outcomes of activities and participation in children and adults born preterm without cerebral palsy: A systematic review
Source: Dev Med Child Neurol. 2026 Mar 27;68(9):1207–27. doi: 10.1111/dmcn.70242 (PMC13436715; doi:10.1111/dmcn.70242)
Supplement: Supplementary file 4 [file DMCN-68-1207-s005.docx]

**Table S4.** Characteristics of the included articles reporting associations between motor skills and outcomes of activities and participation of the ICF in alphabetical order.

| Citation | Setting | Design | Criteria preterm group | Statement on CP | Term-born control group^a/b^ | Birth year | Measure of motor skills | Outcome of association | Measure of outcome | ICF Chapter | Analysis of association | Confounders^c^ |
| --- | --- | --- | --- | --- | --- | --- | --- | --- | --- | --- | --- | --- |
| Aubert et al. 2023^1^ | Europe | Cohort | GA<32 wks | Separate results for CP | No | 2011-2012 | Movement ABC-2 | HRQoL | PedsQL | 9 | Linear regression | Age, sex, family sociodemographic factors |
| Benassi et al. 2016^2^ | Italy | Cohort | GA≤28 wks | No major cerebral damage | Yes/Yes | n.r. | Bayley-III Motor | Communication | Video-recorded gestures | 3 | Correlation | No |
| Blasco et al. 2023^3^ | USA | Cross-sectional | BW<2500g | Children with CP attended another  clinic | No | n.r. (assessed 2015-2018) | Bayley-III Motor | General competence, Socialization | DMQ18 | 2, 7 | Correlation | No |
| Bogičević & van Baar 2019^4^ | Netherlands | Cohort | GA=32-36 wks | No statement | Yes/Yes | 2010-2011 | Bayley-III Motor | Attention, Behavior | CBCL | 1, 2 | Linear regression | Maternal education, cognitive and language skills |
| Bolk et al. 2018^5^ | Sweden | Cohort | GA<27 wks | Separate results for CP | Yes/No | 2004-2007 | Movement ABC-2 | Attention,  Behavior,  Social skills | Brown ADD Scales, SDQ, FTF | 1, 2, 7 | Logistic regression | Sex, GA, age, non-Nordic origin of mothers, twins |
| Brown et al. 2023^6^ | Australia | Cohort | GA<30 wks or BW<1250g | Analyses excluding CP | Yes/Yes | 2001-2003 | Movement ABC-2 | Attention | TEA, SDQ | 1 | Linear regression | Sex, social risk |
| Charkaluk et al. 2019^7^ | France | Cohort | GA<35 wks | CP excluded | No | 2011 | ASQ-2 Fine and Gross Motor | Language | CDI | 3 | Linear mixed model | Sex, GA, growth restriction, maternal parity, maternal education, home language, multiple births |
| Danks et al. 2017^8^ | Australia | Cohort | BW<1000g | CP excluded | Yes/Yes | 1992-1994 | Movement ABC | Behavior | CBCL | 2 | Linear regression | Sex, SGA, SES |
| Davis et al. 2007^9^ | Australia | Cohort | GA<28 wks or BW<1000g | CP excluded | Yes/No | 1991-1992 | Movement ABC | Reading,  Spelling,  Mathematics,  Behavior | WRAT, BASC | 1, 2 | Student’s t-test | Sex, perinatal factors |
| Engan et al. 2020^10^ | Norway | Cohort | GA<28 wks | CP excluded | Yes/No | 1999-2000 | Movement ABC | Physical activity,  Sports participation | Questionnaire | 5, 9 | Logistic regression | Maternal education, asthma medication |
| FitzGerald et al. 2024^11^ | Australia | Cohort | GA<30 wks | CP excluded | No | 2011-2013 | Movement ABC-2 | Physical activity | Axivity AX3 | 5 | Linear regression | Twin births |
| Greenberg & Crnic 1988^12^ | USA | Cohort | GA<38 wks (range 27-36) and BW<1801g | No major identifiable abnormalities | Yes/Yes | 1983 | BSID | Behavior | Parent-report, infant behavior and interaction | 2 | Linear regression | No |
| Hasler & Akshoomoff 2019^13^ | USA | Cohort | GA<33 wks | Moderate-severe CP excluded | Yes/Yes | n.r. | Movement ABC-2 | Mathematics | TEMA-3 | 1 | Mediation analysis | Cognitive measures |
| Helin et al. 2022^14^ | Finland | Cohort | GA<37 wks and BW≤1500g | Separate results for CP | No | 2001-2006 | Movement ABC-2 | Loneliness, Social competence | PNDL, MASCS | 7 | Correlation | Sex |
| Holsti et al. 2002^15^ | Canada | Cohort | BW<800g | CP excluded | No | 1982-1987 | BOTMP | Reading,  Spelling  Mathematics | WRAT | 1 | Logistic regression | No |
| Howe et al. 2016^16^ | Taiwan | Cohort | GA<32 wks and BW<1500g | CP excluded | No | n.r. (recruited at 5 years in 2007-2009) | AIMS,  BSID PDI | Communication,  Daily living skills,  Socialization | VABS-C | 3, 5, 7 | Linear regression | Sex, intrauterine growth, neonatal status, maternal education |
| Jeyaseelan et al. 2006^17^ | Australia | Cohort | GA>27 wks or BW<1000g | CP excluded | No | 1992-1995 | NSMDA | Attention | CRSR, ADHD-RS | 1 | Linear regression | Sex, GA, BW, public/private admission, maternal age, maternal education |
| Jongmans et al. 1997^18^ | UK | Cohort | GA<35 wks | Separate results for CP | No | 1984-1986 | Movement ABC | Behavior | Rutter Scales | 2 | Correlation | No |
| Korkman et al. 2008^19^ | Finland | Cohort | BW<1000g | Separate results for CP | No | 1996-1997 | Modified Touwen test Motor Coordination | Attention and executive function, Language | NEPSY | 1, 3 | MANOVA | SGA, manual motor function |
| Liu et al. 2021^20^ | Germany | Cohort | GA<32 wks or BW<1500g | No statement | Yes/Yes | 1985-1986 | TOMI | Social self-concept | PSPCSA, SCA | 7 | Mediation analysis | Sex, SES |
| Lowe et al. 2019^21^ | USA | Cohort | GA<27 wks or BW<1000g | No statement | No | 2014-2016 | Bayley-III Motor | Attention,  Behavior | CBCL | 1, 2 | Linear regression | Sex, GA, SGA, age, neonatal factors, center, maternal factors |
| Maggi et al. 2014^22^ | Brazil | Cross-sectional | GA≤34 wks and BW≤1500g | No evidence of CP | Yes/No | n.r. | Movement ABC-2 | Daily living | PEDI-FS, PEDI-CA | 5 | Kruskal-Wallis, regression | GA, BW, peri-intraventricular hemorrhage, bronchopulmonary dysplasia |
| Marlow et al. 1993^23^ | UK | Cohort | BW≤1250g | CP excluded | Yes/No | 1980-1981 | TOMI | Academic performance | School difficulties | 8 | Cross tabulations | SES |
| Martel et al. 2007^24^ | USA | Cohort | BW≤2500g | Severe neurologic impairment excluded | Yes/Yes | 1983-1985 | GP | Attention | CBCL | 1 | Mediation analysis | Sex, arousal |
| Moreira et al. 2014^25^ | Brazil | Cross-sectional | GA<35 wks or BW<1500g | No statement | No | 2002-2004 | Movement ABC-2 | Behavior,  Academic performance | SDQ, TDE, parent-reports | 2, 8 | Chi-square test | Sociodemographic characteristics |
| Panceri et al. 2022^26^ | Brazil | Cohort | GA<32 wks or BW<1500g | No statement | No | n.r. | Bayley-III Motor | Language | Bayley-III Language | 3 | Correlation | Maternal care routine, mother knowledge of  child development, home opportunities for development |
| Poole et al. 2015^27^ | Canada | Cohort | BW<1000g | Analyses adjusted for NSI including CP | Yes/Yes | 1977-1982 | ADC | Attention | CBCL, YASR, Barkley Adult ADHD Rating scale | 1 | Linear regression | Neurosensory impairments |
| Proulx et al. 2017^28^ | Canada | Cross-sectional | ≤29 weeks | Adjusted for health problems, including CP | No | 1991-2000 | Movement ABC-2 | Physical activity | ActiGraph GTX3 | 5 | Linear regression | Sex, age, health problems, hyperactivity behavior |
| Roberts et al. 2011^29^ | Australia | Cohort | GA<28 wks or BW<1000g | CP excluded | Yes/No | 1991-1992, 1997 | Movement ABC | Reading, Spelling, Arithmetic | WRAT-3 | 1 | Student’s t-test | No |
| Spittle et al. 2022^30^ | Australia | Cohort | <30 weeks | CP excluded | No | n.r. | Movement ABC-2 | Behavior,  Daily living skills, Social functioning, HRQoL | SDQ, PEDI-CAT,  PedsQL | 2, 5, 7, 9 | Linear regression | Sex, GA, BW, neonatal factors |
| Uusitalo et al. 2020^31^ | Finland | Cohort | GA<32 wks (BW≤1500g in 2001-2003) | Separate results for CP | No | 2001-2006 | Movement ABC-2 | HRQoL | 17D | 9 | Mann-Whitney U test | BW, GA, parental education |
| van Dokkum et al. 2021^32^ | Netherlands | Cohort | GA<36 wks | No statement | No | n.r. | Age of walking | Problem-solving, Communication, Personal-Social | ASQ | 2, 3, 5 | Logistic regression | Sex, SGA, maternal education, ethnicity |
| Verkerk et al. 2014^33^ | Netherlands | Cohort (follow-up RCT) | GA<32 wks or BW<1500g | CP excluded | Yes/No | 2004-2007 | VMI-6 Motor Coordination | Educational provision | Receiving learning support | 8 | Logistic regression | Cognitive measures, executive function, behavior |
| Wiingreen et al. 2025^34^ | Denmark | Cohort | GA<28 wks or BW<1000g | Analysis excluding CP | Yes/No | 1994-1995 | Movement ABC-2 | School difficulties | Failed final examination or GPA≤4.0 in language and mathematics | 8 | Logistic regression | Sex, maternal education, multiple births, intelligence quotient, motor performance |
| Wocadlo & Rieger 2008^35^ | Australia | Cohort | <30 weeks | CP excluded | No | 1987-1997 | BOTMP | Academic achievement,  School difficulties,  Sports participation | WIAT, NARA, SAST, TEMA, Remedial education, Sports activity | 1, 8, 9 | Chi-square test | No |
| Wollum et al. 2022^36^ | Norway | Cohort | ≤1500g | Similar results when CP excluded | Yes/No | 1986-1988 | BSID PDI, PDMS, Movement ABC | Adaptive and general functioning, HRQoL | ASR, GAF, SF36 | 2, 9 | Linear regression | Sex, SES |

^a^Study included a term-born control group

^b^Study examined associations between motor skills and activity/participation outcome in a term-born control group

^c^Factors adjusted for in association analyses or considered as confounders of motor skills or activity/participation outcome

Abbreviations: ADC, Adult Developmental Coordination Disorders/Dyspraxia Checklist; ADD, Attention Deficit Disorder; ADHD, Attention Deficit Hyperactivity Disorder; ADHD-RS, Attention Deficit Hyperactivity Disorder Rating Scale; AIMS, Alberta Infant Motor Scale; ASR, Adult Self-Report; ASQ, Ages & Stages Questionnaire; ASQ-2, Ages & Stages Questionnaire, Second Edition; BASC, Behavior Assessment System for Children; BNBAS, Brazelton Neonatal Behavioral Assessment Scale; BOTMP, Bruininks-Oseretsky Test of Motor Proficiency; BSID, Bayley Scales of Infant Development; Bayley-III, Bayley Scales of Infant and Toddler Development, Third Edition; BW, birth weight; CAPE, Children’s Assessment of Participation and Enjoyment; CBCL, Child Behavior Checklist; CDI, MacArthur-Bates Communicative Development Inventories; CI, confidence interval; CP, cerebral palsy; CRSR, Conners’ Rating Scale Revised-Long Form; CSEI, Coopersmith Self-Esteem Inventory; DMQ18, Revised Dimensions of Mastery Questionnaire; FTF, Five to Fifteen Questionnaire; g, grams; GA, gestational age; GAF, Global Assessment of Functioning; GP, Grooved Pegboard; GPA, Grade Point Average; GSW Harter Self-Perception Profile for Adolescents Global Self-Worth subscale; HRQoL, health-related quality of life; ICF, International Classification of Functioning, Disability and Health; MANOVA, Multivariate analysis of variance; Movement ABC, Movement Assessment Battery for Children; Movement ABC-2, Movement Assessment Battery for Children, Second Edition; MASCS, Multisource Assessment of Children’s Social Competence Scale; NARA, Neale Analysis of Reading Ability; NEPSY, Developmental Neuropsychological Assessment; NSMDA, Neurological, Sensory, Motor, Developmental Assessment; PDI, Psychomotor Development Index; PDMS; Peabody Developmental Motor Scales; PEDI, Pediatric Evaluation of Disability Inventory; PEDI CAT, Pediatric Evaluation of Disability Inventory – Computer Adaptive Test; PedsQL, Pediatric Quality of Life Inventory; PNDL, Peer Network and Dyadic Loneliness scale; PSPCSA, Pictorial Scale of Perceived Competence and Social Acceptance for Young Children; RCT, randomised controlled trial; RSES, Rosenberg Self-Esteem Scale; SAST, South Australian Spelling Test; SCA, Nicholls’ Self-concept of Attainment; SDQ, Strengths and Difficulties Questionnaire; SES, socioeconomic status; SF36, Short Form 36 Health Survey; SGA, small for gestational age; TDE, Teste de Desempenho Escolar; TEA, Test of Everyday Attention for Children; TEMA, Test of Early Mathematics Ability; TEMA-3, Test of Early Mathematics Ability, Third Edition; TOMI, Test of Motor Impairment; YASR, Young Adult Self Report; YC-PEM, Young Children's Participation and Environment Measure; VABS-C, Vineland Adaptive Behavior Scales – Chinese version; VMI-6, Beery-Buktenica Developmental Test of Visual-Motor Integration, Sixth Edition; WIAT, Wechsler Individual Achievement Test; wks, weeks; WRAT, Wide Range Achievement Test; 17D, 17-Dimensional Illustrated Questionnaire.

**References**

1. Aubert AM, Costa R, Johnson S, et al. Developmental motor problems and health-related quality of life in 5-year-old children born extremely preterm: A European cohort study. Dev Med Child Neurol. 2023;65(12):1617–1628.
2. Benassi E, Savini S, Iverson JM, et al. Early communicative behaviors and their relationship to motor skills in extremely preterm infants. Res Dev Disabil. 2016;48:132–144.
3. Blasco P, Saxton S, Gullion LM, et al. Assessment of Mastery Motivation and Neurodevelopment of Young Children at High Risk for Developmental Delays. Journal of Intelligence. 2023;11(6).
4. Bogičević L, Verhoeven M, van Baar AL. Toddler skills predict moderate-to-late preterm born children's cognition and behaviour at 6 years of age. PLoS One. 2019;14(11):e0223690.
5. Bolk J. FA, Hafström M., Åden U., Serenius F. Developmental Coordination Disorder and Its Association With Developmental Comorbidities at 6.5 Years in Apparently Healthy Children Born Extremely Preterm. JAMA pediatrics. 2018;172(8):765–774.
6. Brown RN, Burnett AC, Thompson DK, et al. Motor performance and attention outcomes in children born very preterm. Dev Med Child Neurol. 2023;65(11):1501–1510.
7. Charkaluk ML, Rousseau J, Benhammou V, et al. Association of Language Skills with Other Developmental Domains in Extremely Very and Moderately Preterm Children: EPIPAGE 2 Cohort Study. The Journal of pediatrics. 2019;208:114.
8. Danks M, Cherry K, Burns YR, et al. Are behaviour problems in extremely low-birthweight children related to their motor ability? Acta paediatrica (Oslo Norway : 1992). 2017;106(4):568–572.
9. Davis NM, Ford GW, Anderson PJ, et al. Developmental coordination disorder at 8 years of age in a regional cohort of extremely-low-birthweight or very preterm infants. Dev Med Child Neurol. 2007;49(5):325–330.
10. Engan M, Engeseth MS, Fevang S, et al. Predicting physical activity in a national cohort of children born extremely preterm. Early Hum Dev. 2020;145.
11. FitzGerald TL, Cameron KL, Albesher RA, et al. Associations between physical activity and development in preschool-aged children born <30 weeks' gestation: a cohort study. Arch Dis Child Fetal Neonatal Ed. 2024;109(6):602–608.
12. Greenberg MT, Crnic KA. Longitudinal predictors of developmental status and social interaction in premature and full-term infants at age two. Child Dev. 1988;59(3):554–570.
13. Hasler HM, Akshoomoff N. Mathematics ability and related skills in preschoolers born very preterm. Child Neuropsychol. 2019;25(2):162–178.
14. Helin M, Karukivi M, Haataja L, et al. Motor Performance in Association with Perceived Loneliness and Social Competence in 11-Year-Old Children Born Very Preterm. Children. 2022;9(5).
15. Holsti L, Grunau RVE, Whitfield MF. Developmental coordination disorder in extremely low birth weight children at nine years. J Dev Behav Pediatr. 2002;23(1):9–15.
16. Howe TH, Sheu CF, Hsu YW, et al. Predicting neurodevelopmental outcomes at preschool age for children with very low birth weight. Res Dev Disabil. 2016;48:231–241.
17. Jeyaseelan D, O'Callaghan M, Neulinger K, et al. The association between early minor motor difficulties in extreme low birth weight infants and school age attentional difficulties. Early Hum Dev. 2006;82(4):249–255.
18. Jongmans M, Mercuri E, De Vries L, et al. Minor neurological signs and perceptual-motor difficulties in prematurely born children. Arch Dis Child Fetal Neonatal Ed. 1997;76(1):F9–F14.
19. Korkman M, Mikkola K, Ritari N, et al. Neurocognitive test profiles of extremely low birth weight five-year-old children differ according to neuromotor status. Dev Neuropsychol. 2008;33(5):637–655.
20. Liu Y, Mendonça M, Bartmann P, et al. Very preterm birth and trajectories of domain-specific self-concept from childhood into adulthood. Dev Psychopathol. 2021:1–12.
21. Lowe JR, Fuller JF, Do BT, et al. Behavioral problems are associated with cognitive and language scores in toddlers born extremely preterm. Early Hum Dev. 2019;128:48–54.
22. Maggi EF, Magalhães LC, Campos AF, et al. Preterm children have unfavorable motor cognitive and functional performance when compared to term children of preschool age. J Pediatr (Rio J). 2014;90(4):377–383.
23. Marlow N, Roberts L, Cooke R. Outcome at 8 years for children with birth weights of 1250 g or less. Arch Dis Child. 1993;68(3 SUPPL.):286–290.
24. Martel MM, Lucia VC, Nigg JT, et al. Sex differences in the pathway from low birth weight to inattention/hyperactivity. J Abnorm Child Psychol. 2007;35(1):87–96.
25. Moreira RS, Magalhães LC, Dourado JS, et al. Factors influencing the motor development of prematurely born school-aged children in Brazil. Res Dev Disabil. 2014;35(9):1941–1951.
26. Panceri C, Silveira RC, Procianoy RS, et al. Motor development in the first year of life predicts impairments in cognition and language at 3 years old in a Brazilian preterm cohort of low-income families. Front Neurosci. 2022;16.
27. Poole KL, Schmidt LA, Missiuna C, et al. Motor coordination and mental health in extremely low birth weight survivors during the first four decades of life. Res Dev Disabil. 2015;43-44:87–96.
28. Proulx K, Majnemer A, Dahan-Oliel N, et al. Factors Associated With Moderate to Vigorous Physical Activity in Adolescents Born Preterm. Pediatr Exerc Sci. 2017;29(2):260–267.
29. Roberts G, Anderson PJ, Davis N, et al. Developmental coordination disorder in geographic cohorts of 8-year-old children born extremely preterm or extremely low birthweight in the 1990s. Dev Med Child Neurol. 2011;53(1):55–60.
30. Spittle AJ, Olsen JE, FitzGerald TL, et al. School Readiness in Children Born <30 Weeks' Gestation at Risk for Developmental Coordination Disorder: A Prospective Cohort Study. J Dev Behav Pediatr. 2022;43(5):E312–E319.
31. Uusitalo K, Haataja L, Nyman A, et al. Preterm children's developmental coordination disorder cognition and quality of life: a prospective cohort study. BMJ Paediatrics Open. 2020;4(1):e000633.
32. van Dokkum NH, Reijneveld SA, Bos AF, et al. Attainment of smiling and walking in infancy associates with developmental delays at school entry in moderately-late preterm children: a community-based cohort study. BMC Pediatr. 2021;21(1):84.
33. Verkerk G, Jeukens-Visser M, van Wassenaer-Leemhuis A, et al. The relationship between multiple developmental difficulties in very low birth weight children at 3½ years of age and the need for learning support at 5 years of age. Res Dev Disabil. 2014;35(1):185–191.
34. Wiingreen R, Greisen G, Esbjorn BH, et al. Five-year neurodevelopmental assessment of extremely preterm or extremely low birthweight infants: Association with school performance. Acta Paediatr. 2025;114(1):131–140.
35. Wocadlo C, Rieger I. Motor impairment and low achievement in very preterm children at eight years of age. Early Hum Dev. 2008;84(11):769–776.
36. Wollum AEK, Berdal EK, Iversen JM, et al. Outcomes and predictors of functioning mental health and health-related quality of life in adults born with very low birth weight: a prospective longitudinal cohort study. BMC Pediatr. 2022;22(1):628.
